# The multifaceted role of autophagy in cancer and the microenvironment

**DOI:** 10.1002/med.21531

**Published:** 2018-10-09

**Authors:** Hendrik Folkerts, Susan Hilgendorf, Edo Vellenga, Edwin Bremer, Valerie R. Wiersma

**Affiliations:** ^1^ Department of Hematology Cancer Research Center Groningen, University Medical Center Groningen, University of Groningen Groningen The Netherlands

**Keywords:** autophagy, cancer, immune cells, microenvironment, stroma, therapy

## Abstract

Autophagy is a crucial recycling process that is increasingly being recognized as an important factor in cancer initiation, cancer (stem) cell maintenance as well as the development of resistance to cancer therapy in both solid and hematological malignancies. Furthermore, it is being recognized that autophagy also plays a crucial and sometimes opposing role in the complex cancer microenvironment. For instance, autophagy in stromal cells such as fibroblasts contributes to tumorigenesis by generating and supplying nutrients to cancerous cells. Reversely, autophagy in immune cells appears to contribute to tumor‐localized immune responses and among others regulates antigen presentation to and by immune cells. Autophagy also directly regulates T and natural killer cell activity and is required for mounting T‐cell memory responses. Thus, within the tumor microenvironment autophagy has a multifaceted role that, depending on the context, may help drive tumorigenesis or may help to support anticancer immune responses. This multifaceted role should be taken into account when designing autophagy‐based cancer therapeutics. In this review, we provide an overview of the diverse facets of autophagy in cancer cells and nonmalignant cells in the cancer microenvironment. Second, we will attempt to integrate and provide a unified view of how these various aspects can be therapeutically exploited for cancer therapy.

## 1 INTRODUCTION

Autophagy is an important homeostatic process in the human body that is responsible for the elimination of damaged and/or superfluous macromolecules such as proteins and lipids as well as the removal of damaged organelles like mitochondria. The successful execution of autophagy enables the recycling of nutrients, amino acids, and lipids and acts as a quality control mechanism to maintain organelle function.^1^, ^2^, ^3^, ^4^ The importance of autophagy is evidenced by the fact that a block in autophagic flux due to knockdown of core autophagy genes is detrimental during the early development in murine models.^5^, ^6^, ^7^, ^8^, ^9^, ^10^, ^11^ Perhaps not surprisingly, an increasing body of evidence highlights the important and multifaceted impact of autophagy in cancer. For instance, during tumor development, the autophagic process appears to function as a tumor suppressor and limits tumorigenesis.^12^, ^13^, ^14^, ^15^ In this respect, it is noteworthy that a single‐nucleotide polymorphism in the promoter region of the crucial autophagy‐related gene (ATG) ATG16L1, which putatively downregulates its expression level, associated with susceptibility to thyroid and colorectal cancer and has a significant negative impact on patient survival in local and advanced metastatic prostate cancer.^16^, ^17^, ^18^ Further, survival of patients with advanced lung adenocarcinoma upon epidermal growth factor receptor (EGFR) tyrosine kinase inhibitor treatment is significantly impacted by functional genetic polymorphisms in core autophagy genes, thus highlighting the potential clinical impact of autophagic signaling on cancer development and response to therapy.^19^

In established cancers, autophagy activity is upregulated during treatment and associated with resistance to cancer therapy.^20^ Further, elevated autophagy maintains stemness in cancer stem cells (CSCs). Moreover, cancer cells appear to rely more on autophagy for continued survival than normal cellular counterparts. Consequently, the inhibition of autophagy is being explored for cancer therapy particularly in combination with other cytotoxic drugs to augment cytotoxicity.^21^, ^22^, ^23^ Autophagy occurring in the context of cancer therapy may, on the one hand, be a stress response that enables cancer cells to survive and evade apoptotic elimination.^4^ In this setting, inhibition of autophagy sensitizes cells to apoptotic cell death and may be of use to augment the efficacy of anticancer agents. On the contrary, autophagy may also be a driver of cytotoxic cell death and in this case inhibition of autophagy would inhibit cell death. This type of cell death has been termed autophagic cell death (ACD) and has been reported, eg, for radiation therapy.^24^, ^25^, ^26^, ^27^, ^28^ Thus, depending on the type of cell death inhibition of autophagy may be warranted for combination therapy.

It is evident that autophagy is more and more emerging as a potential target for cancer therapy. However, the complex microenvironment of an established tumor comprises many different cell types in addition to malignant cells that all to a different extent utilize and rely on the autophagic process. Indeed, as will be discussed in this review, autophagy not only clearly impacts on cancer (stem) cells, but also on stromal cells, endothelial cells, and (tumor‐infiltrated) innate and adaptive immune cells. Therefore, it is crucial to understand the impact of autophagy and its therapeutic targeting in the context of this diverse cellular composition of the tumor microenvironment.

**Figure 1:**
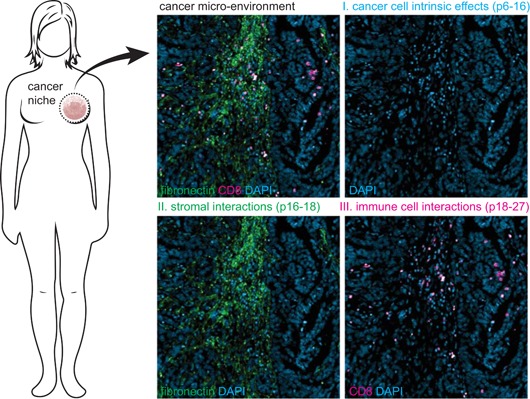
Review outline. This review highlights the impact of changes in autophagy within cancer cells, as well as in the context of the complex cancer microenvironment. Part I describes how aberrant autophagy can contribute to cancer initiation and maintenance as well as therapy resistance (pp. 6‐16). Part II describes the role of autophagy in different stromal cells within the tumor microenvironment, such as fibroblasts and mesenchymal stem cells (pp. 16‐18). Further, the impact of autophagy on anticancer immune responses is described (pp. 18‐27). Blue, 4′,6‐diamidino‐2‐phenylindole (DAPI) staining; green, fibronectin staining for stroma; red, CD8 staining for cytotoxic T‐cell [Color figure can be viewed at wileyonlinelibrary.com]

In this review, we will first briefly detail the core autophagy machinery and regulatory pathways after which we will provide an overview of current thinking on the role of autophagy in cancer cells and the functioning of the diverse components within the tumor microenvironment (illustrated in Figure 1). Further, we will provide directions for incorporating the sometimes opposing effects of autophagy on tumor microenvironmental components for the future implementation of autophagy‐targeting drugs in cancer.

## 2 AUTOPHAGY SIGNALING AND REGULATORY PATHWAYS

The term autophagy defines a process that can occur in three different forms, with the most prominent form being macroautophagy, a form of autophagy that includes removal of proteins and/ or organelles. In the case of mitochondria, this process is called mitophagy. Second, when molecules that have to be degraded are directly invaginated by the lysosome, this process is called microautophagy. Third, proteins can be degraded via chaperone‐mediated autophagy (CMA). During CMA, proteins are targeted for degradation by heat shock protein hsc70 via their KFERQ‐like motif.^29^, ^30^ Unless specifically referred to, the term autophagy in this review describes macroautophagy. In the section below, we will detail basic autophagy pathways as well as highlight regulatory hubs that are important in cancer.

### 2.1 The core autophagy machinery

**Figure 2:**
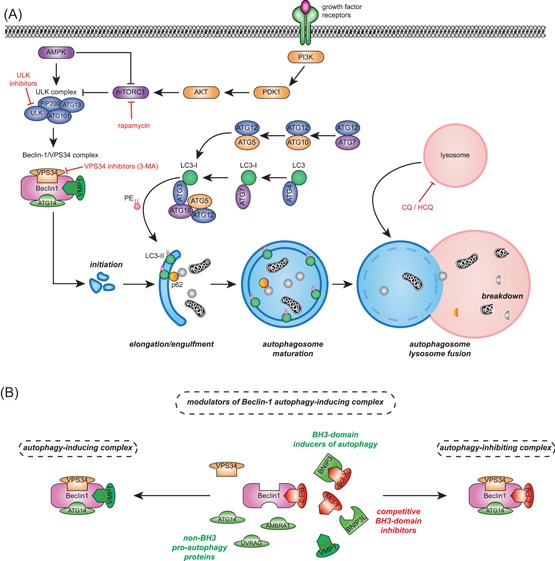
The autophagy pathway. A, The activation of autophagy is initiated by the reduced activity of the mechanistic target of rapamycin complex 1 (mTORC1) complex due to activated adenosine monophosphate‐activated protein kinase (AMPK) or decreased upstream growth signaling. mTORC1 is an inhibitor of the ULK complex, therefore reduced mTORC1 activity increases the activity of the ULK complex. The ULK complex together with the Beclin‐1/ VPS34 complex initiates the formation of autophagosomes. Dependent on the complex composition, Beclin‐1 can act as a molecular switch between autophagy and apoptosis (see B). The expansion and maturation of the autophagosomes is dependent on two ubiquitin‐like conjugation systems, which requires multiple autophagy proteins. First, ATG12‐ATG5 conjugate binds to ATG16, which stimulates LC3 lipidation. Second, LC3 is covalently conjugated to phosphatidylethanolamine (PE) generating LC3‐II, which is incorporated in the autophagosomal membrane. Incorporated LC3‐II is required for binding and internalization of adaptor proteins such as p62. Finally, the mature autophagosome fuses with lysosomes, after which its content is broken down by digestive enzymes. Indicated in red are pharmacological agents, chloroquine (CQ), hydroxychloroquine (HCQ), 3‐methyladenine (3‐MA), and ULK inhibitors, that inhibit autophagy. In addition, rapamycin activates autophagy by inhibiting mTORC1. B, Beclin‐1 is a core member of the VPS34/Beclin‐1 complex, which acts as a molecular switch in controlling autophagy downstream of the ULK1 complex. Depicted in red are the antiapoptotic members of the Bcl‐2 family BCL‐2, BCL‐XL, and MCL‐1 which can bind to Beclin‐1, through interaction with its BH3 domain, thereby inhibiting autophagy. Alternatively, Bcl‐2 interacting protein 3 (BNIP3) and Bcl‐2 interacting protein 3 like (BNIP3L; depicted in green) can competitively bind to antiapoptotic BLC‐2 members. Dissociation of antiapoptotic Bcl‐2 members from Beclin‐1, consequently activates autophagy. Other non‐BH3 proteins, also depicted in green, such as vacuole membrane protein 1 (VMP1), ATG14, UV radiation resistance‐associated gene (UVRAG), and activating molecule in Beclin‐1‐regulated autophagy protein 1 (AMBRA1) can also bind Beclin‐1, thereby activating autophagy. PDK1, pyruvate dehydrogenase kinase 1; PI3K, phosphoinositide 3‐kinase [Color figure can be viewed at wileyonlinelibrary.com]

The execution of autophagy can be subdivided into the initiation phase, elongation phase, autophagosome maturation, autophagosome‐lysosome fusion, and degradation of content in autophagolysosomes (Figure 2A). The initiation of autophagy generally starts at the mechanistic target of rapamycin (mTOR) complex 1 (mTORC1), the master regulator of autophagy, which under basal conditions represses the autophagy pathway by inhibiting the Unc‐51 like autophagy activating kinase 1 (ULK1) complex.^31^ However, upon increased nutrient demand or nutrient limiting conditions, mTORC1 is deactivated due to reduced upstream signaling from the phosphoinositide 3‐kinase (PI3K)/Akt and the mitogen‐activated protein kinase (MAPK) pathway, thereby enabling initiation of autophagy. In addition, the 5′‐adenosine monophosphate‐activated protein kinase (AMPK), a key kinase regulating cellular energy homeostasis, activates the ULK1 complex and inactivates mTORC1 when low energy levels are detected.^19^, ^32^ The activated ULK1 complex, together with the Beclin‐1‐VPS34 complex (a complex discussed in more detail in Section 2.2) initiates the formation of autophagosomes. The formation of autophagosomes can be inhibited by 3‐methyladenine (3‐MA), an inhibitor of VPS34. In contrast, rapamycin, an inhibitor of mTORC1, is generally used as an autophagy inducer. Of note, although mTOR and its complexes have many more functions besides regulating the autophagy pathway, eg, regulation of cell growth, proliferation, protein translation, and metabolism, the inhibition of mTOR is in this review generally used as an activator of autophagy.

Maturation of the autophagosome requires two ubiquitin‐like conjugation systems. First, ATG12 is covalently bound to ATG5, a process mediated by ATG7 and ATG10. The ATG12‐ATG5 conjugate is subsequently noncovalently connected to ATG16, which is required for the localization of ATG12 and ATG5 to the forming autophagosome.^33^ Second, LC3 is converted into LC3‐II, which starts with the proteolytic cleavage of LC3 by ATG4 to form LC3‐I. LC3‐I is then bound by ATG7, which transfers LC3‐I to ATG3.^34^, ^35^, ^36^ ATG3 subsequently catalyzes the conjugation of the lipid phosphatidylethanolamine (PE) to LC3‐I, thereby yielding LC3‐II. This lipidation step is enhanced by the ATG5/ATG12/ATG16 complex. Eventually, LC3‐II is inserted in the membrane of the elongating autophagosome. During the maturation of the autophagosome, proteins and organelles to be degraded are sequestered to the forming autophagosome by p62/sequestosome 1 (SQSTM1). For this purpose, p62 can directly interact with LC3.^37^ Finally, the mature autophagosome fuses with a lysosome to form the autolysosome. The lysosome‐associated membrane proteins (LAMP‐1 and LAMP‐2) are essential for this fusion and also maintain the integrity of lysosomal membranes.^38^ The macromolecules and organelles that have been entrapped in the autophagosomes are then degraded by the digestive enzymes of the lysosomes (eg, lipases, proteases, nucleases, sulfatases), which yields amino acids, fatty acids, and nucleotides for eventual reuse. The fusion of autophagosomes with lysosomes can be inhibited by chloroquine (CQ) or hydroxychloroquine (HCQ), both compounds that prevent acidification of the lysosomes.

Of note, the generation of LC3‐II is considered as a hallmark marker of autophagy induction, whereas its sustained accumulation is reflective of autophagy inhibition.^39^ In addition, p62 is degraded during the proper execution of autophagy, and its accumulation can be used as a marker for inhibition of autophagy.^40^

### 2.2 Bcl‐2 family members modulate Beclin‐1‐dependent autophagy

Beclin‐1 is an important regulatory hub to which proautophagic and antiautophagic proteins can bind (Figure 2B). First, the antiapoptotic proteins of the BCL‐2 family, eg, BCL‐2, BCL‐XL, and MCL‐1, can bind to the characteristic BH3 domain of Beclin‐1, which inhibits autophagy.^41^, ^42^, ^43^ Second, non‐Bcl‐2 family proteins like UV radiation resistance‐associated gene (UVRAG), activating molecule in Beclin‐1‐regulated autophagy protein 1 (AMBRA1), high mobility group box 1 (HMGB1), and vacuole membrane protein 1 (VMP1) can competitively bind to Beclin‐1 at the same domain, which can shift the balance to induction of autophagy.^44^, ^45^, ^46^, ^47^ In addition, the hypoxia‐inducible Bcl‐2 interacting protein 3 (BNIP3) and Bcl‐2 interacting protein 3 like (BNIP3L) proteins that also contain a BH3 domain can directly interact with BCL‐2 family members.^48^ This BNIP3‐Bcl‐2 interaction prevents Bcl‐2 binding to Beclin‐1 and, thereby, promotes autophagy. Alterations in the pool of Beclin‐1 interacting proteins can alter the balance of autophagy regulation. In line with this, gene silencing of Bcl‐2 using small interfering RNA (siRNA) in MCF‐7 cells triggered autophagy, whereas in neuron‐specific MCL‐1‐knockout mice autophagy was increased in neuronal cells.^49^, ^50^ Correspondingly, treatment of various cancer cell lines with BH3 mimetics that promote dissociation of Bcl‐2 or BCL‐XL from Beclin‐1‐activated autophagy.^51^, ^52^ Here, autophagy was inhibited by siRNA‐mediated knockdown of essential autophagy proteins.^53^ In a recent screen, three compounds were identified that specifically disrupt the binding between BCL‐2 and Beclin‐1.^54^ These compounds de‐repressed autophagy without causing any cytotoxicity.^54^ The induction of mitophagy can also be regulated by Bcl‐2 members. In brief, mitochondrial depolarization promoted Parkin and PTEN‐induced putative kinase 1 (PINK1)‐dependent induction of mitophagy, which was suppressed by transient overexpression of Bcl‐2 family members MCL‐1 and BLC‐XL.^55^, ^56^ In this case, inhibition of mitophagy was independent of Beclin‐1, but due to inhibition of Parkin translocation to depolarized mitochondria.^55^ Taken together, the elevated expression of members of the BCL‐2 family can reduce autophagy, including mitophagy.

## 3 PART I: THE ROLE OF AUTOPHAGY IN CANCER CELLS

**Figure 3:**
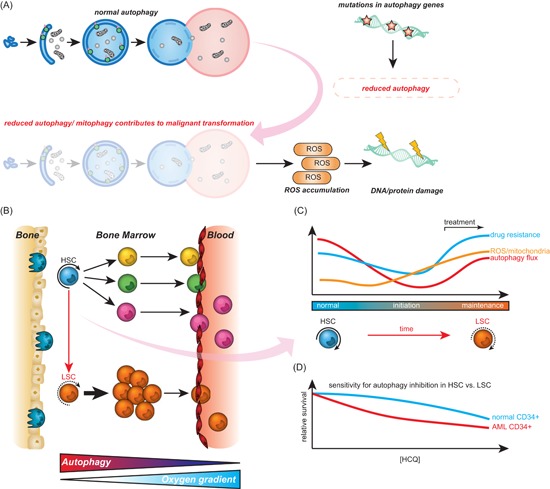
Autophagy during malignant transformation and cancer maintenance. A, Different pro‐oncogenic events such as mutation or monoallelic deletion of autophagy‐related genes can cause reduced autophagy activity. Reduced levels of autophagy/mitophagy can contribute to malignant transformation due to elevated levels of reactive oxygen species (ROS). B, Hematopoietic stem cells (HSCs) reside in specific bone marrow niches with low oxygen content and are characterized by high autophagy activity. During differentiation, the autophagy flux declines and mature cells leave the bone marrow (BM) environment and enter the blood‐stream. In leukemia, HSCs have acquired mutations which results in a block in differentiation and consequently accumulation of immature blasts in BM and peripheral blood of patients. C, Hypothetical model for changes in autophagy and ROS in HSCs during transformation. Normal HSCs have high autophagy flux, low mitochondrial activity, and ROS levels. During cancer initiation, autophagy is repressed (although not completely inhibited), causing accumulation of mitochondria and ROS, which in turn contributes to malignant transformation. During cancer maintenance, cancer cells re‐establish functional autophagy promoting tumor growth and survival. In addition, in response to drug treatment, autophagy is activated and acts as a survival mechanism for cancer cells. D, Both normal BM‐derived CD34^+^ and acute myeloid leukemia (AML) CD34^+^ cells need a certain level of autophagy to survive. Therefore, there is only a small therapeutic window of autophagy inhibition with autophagy inhibitors like hydroxychloroquine. LSC, leukemic stem cells [Color figure can be viewed at wileyonlinelibrary.com]

Autophagy has a multifactorial impact on cancer and influences both cancer initiation and maintenance, as well as regulates cancer response to therapy. Alterations in autophagy levels due to mutations in key autophagy genes or aberrant activation of autophagy regulators have been associated with tumorigenesis (illustrated in Figure 3A). In this respect, cancer initiation is associated with reduced autophagy levels, which leads to the accumulation of oncogenes and reactive oxygen species (ROS). In contrast, during cancer maintenance, the activity of the autophagy pathway is often upregulated. This upregulation ensures sufficient energy supply and contributes to survival during stress, eg, hypoxia and metastasis (illustrated in Figure 3B). During anticancer therapy, autophagy is increased by which cancer cells survive and gain therapy resistance. In addition, CSCs appear to rely on autophagy to maintain stemness.

### 3.1 Impact of autophagy in early tumorigenesis

Autophagy is likely important for cancer initiation as mice with monoallelic deletion of the key autophagy regulator Beclin‐1 have an increased susceptibility to spontaneous tumor development.^13^ In line with this, monoallelic deletions of Beclin‐1 have been detected in human breast cancer, prostate, and ovarian cancer, whereas reduced expression of Beclin‐1 was detected in brain cancer.^57^, ^58^, ^59^, ^60^, ^61^ Similarly, monoallelic deletion of other essential autophagy genes such as ATG5, ATG7, or total loss of ATG4C have been associated with an increased risk of developing malignancies.^14^, ^15^ Based on this data autophagy appears to act as a tumor suppressor with reduced levels of autophagy associating with an accumulation of dysfunctional organelles and proteins that may contribute to malignant transformation. Of note, a low constitutive level of autophagy is required for cell survival, as evidenced by the fact that the knockout of ATG genes, Beclin‐1, or AMBRA1 is embryonically lethal in mice.^13^, ^62^ As described in more detail below, there are several mechanisms in cancer that can reduce autophagic flux, eg mutations in core autophagy genes that may trigger cancer development. These processes and their potential impact on cancer initiation are reviewed in more detail below.

#### 3.1.1 Mutations in autophagy genes that affect autophagy levels during tumor development

Alterations in expression of various key autophagy genes have been reported for different types of cancer, including breast, lung, pancreatic, bladder cancer, and leukemia.^63^ As mentioned above, one of the common molecular aberrations is the loss of one of the alleles of the essential autophagy gene Beclin‐1. This aberration was detected in subsets of cancers, even in breast carcinoma cell lines that are often polyploid for the Beclin‐1 encoding chromosome 17.^64^, ^65^, ^66^ Interestingly, reduced autophagy due to allelic loss of Beclin‐1 in immortalized mouse kidney cells or mouse mammary epithelial cells led to a profound increase in DNA damage.^67^, ^68^ The increased DNA damage was associated with chromosomal abnormalities that are linked to cancer initiation, such as gene amplification and aneuploidy.^67^, ^68^ For example, in immortalized mouse kidney cells the chromosome number (normally 40) was increased to an average of 56 after allelic loss of *Beclin‐1.* 68 Moreover, mammary tissue in Beclin‐1^+/−^ mice developed benign neoplasia with hyperproliferation, whereas reintroduction of Beclin‐1 expression in breast cancer MCF‐7 cells suppressed tumorigenesis.^66^, ^69^ However, mouse models with loss of Beclin‐1 or other essential autophagy proteins do not develop many different types of cancers.^70^ Also, Beclin‐1 is not specifically mutated or deleted in cancer, but rather lost due to deletions in chromosome 17Q21.^70^ So, it is not completely clear if the loss of Beclin‐1 directly contributes to cancer initiation. Similar to Beclin‐1, allelic loss of the autophagy component UVRAG or reduced expression of Bif‐1, both direct interactors with Beclin‐1, is also associated with cancer development, in this case, gastric and colon cancer.^71^, ^72^, ^73^ In brief, UVRAG forms a complex with Beclin‐1 to activate autophagy and loss of this protein resulted in impaired autophagy. Moreover, UVRAG prevented accumulation of abnormal chromosomes, although it is not clear whether this feature is autophagy dependent.^74^ Bif‐1 interacts with Beclin‐1 and UVRAG and also serves to activate autophagy.^44^ Consequently, loss of Bif‐1 expression reduces autophagy and in knockout mice resulted in an increased number of spontaneous tumors.^44^ Together with the above‐described data on Beclin‐1, these findings suggest that autophagy regulation by Beclin‐1 is an important hub that is deregulated in cancer. Further, disruption of Beclin‐1/UVRAG/BIF‐1 may cause genomic instability.^75^ In addition, GABARAPL1, an autophagy gene involved in of the initiation of autophagosome formation, was found to be downregulated in breast cancer, in this case, due to altered DNA methylation and histone deacetylation patterns.^76^ The functional outcome of downregulation of GABARAPL1 was a reduction in autophagic flux and increased tumorigenesis.^77^

In a recent screening approach, a more detailed picture of the mutational spectrum of 180 autophagy genes was obtained, using whole‐exome sequencing of 223 cases of myeloid neoplasm. Copy number alterations or missense mutations were detected in roughly 22% of autophagy‐associated genes and 14% of the studied cases.^78^ Interestingly, the majority of mutations were nonsynonymous substitutions that associated with adverse prognosis. Clonal hierarchy analysis indicated that these autophagy mutations were predominantly secondary events.^78^ In addition to mutations in core autophagy genes, mutations in the spliceosome that are linked to aberrant autophagy gene expression in myeloid malignancy were also found. For example, the splicing factor U2AF35, which is mutated in ~10% of patients with myelodysplastic syndrome, caused abnormal processing of ATG7 pre‐messenger RNA (pre‐mRNA) and consequently reduced the expression of ATG7.^79^ Interestingly, complete knockout of ATG7 in hematopoietic stem cells (HSCs) in mice causes severe anemia and in the long‐term triggered atypical myeloproliferation and accumulation of myeloid blasts in organs, all characteristics associated with myeloid malignancies.^80^, ^81^, ^82^ How autophagic flux is affected by these mutations remains to be functionally defined, but the likely outcome is a reduction in the level of autophagy. Indeed, the nonsynonymous substitutions observed in leukemia are often hypomorphic, that is mutations that cause reduced expression, suggesting that autophagy is repressed but not completely inhibited.^78^ In line with this, complete inhibition of autophagy due to, eg, bi‐allelic deletions or premature stop codons were not observed in any of the core autophagy genes in myeloid neoplasms.^78^ Further, in cross‐cancer unsupervised clustering analysis, autophagy‐associated transcript levels significantly correlated with overall survival in leukemia, kidney cancer, and endometrial cancer.^83^ Overall, these findings suggest that mutations in autophagy genes are relevant during tumorigenesis, with autophagy generally being downregulated but not lost.

#### 3.1.2 Defective mitophagy causes accumulation of reactive oxygen species

Downregulation of mitophagy, the term used for the autophagic removal of dysfunctional mitochondria, can result in an increase in the formation of ROS.^84^, ^85^, ^86^ Disruption of mitophagy by knockout of essential autophagy genes such as *ATG5*, *ATG7*, *ATG12*, and *FIP200* coincides with accumulation of defective mitochondria and increased ROS levels.^7^, ^87^, ^88^, ^89^ Such oxidative stress has been linked to cancer development and progression.^90^ For instance, persistent accumulation of ROS can damage proteins, fatty acids, and DNA, which may contribute to cancer development.^90^, ^91^, ^92^ Further, protein and lipid phosphatases can be inactivated upon oxidation of cysteine residues in the catalytic domain, causing changes in signaling pathways and affecting cell growth.^93^ Interestingly, the autophagy protein ATG4 is a cysteine protease that is overexpressed in several types of cancer and is highly sensitive to ROS.^94^, ^95^, ^96^ Redox modifications of cysteine residues in ATG4 prevent delipidation of LC3, thereby promoting sustained autophagy.^96^ In human adenocarcinoma cells, oxidative stress led to upregulation of ATG4 together with increased autophagy and increased invasion of cells through a Matrigel matrix.^97^ Another example of the interplay between ROS and autophagy is the accumulation of p62/SQSTM1, a scaffold protein for ubiquitinated cargo that is continuously cleared via basal autophagy.^98^ Accumulation of p62 aggregates due to crippling of autophagy causes oxidative stress and triggers the DNA damage response pathway.^99^ However, elevated ROS levels can also activate p53‐mediated apoptotic cell death.^100^ Of note, mutant p53 was shown to attenuate expression of ROS‐scavenging enzymes coinciding with high ROS levels, indicating that these cells are able to tolerate ROS levels to a higher degree.^101^ The exact interplay between autophagy and ROS in cancer development is highly complex, and it remains unclear how persistent elevation of ROS, due to defective autophagy can contribute to cancer development.

#### 3.1.3 Autophagy prevents accumulation of oncoproteins

Reduced autophagy levels during tumorigenesis may also alter the intracellular levels of oncoproteins. Indeed, several oncoproteins have been shown to be a target for degradation via CMA. For example, BCR‐ABL, an oncoprotein formed by chromosomal translocation, was targeted to the autolysosome by CMA after treatment of chronic myeloid leukemia (CML) cell lines and primary CML patient–derived cells with the chemotherapeutic arsenic trioxide.^102^ In line with this data, inhibition of autophagy prevented arsenic trioxide–mediated suppression of BCR‐ABL expression.^102^ Defective autophagy was similarly associated with accumulation of the oncoprotein PML/RARA, the hallmark oncoprotein of acute promyelocytic leukemia.^103^ Moreover, treatment of acute myeloid leukemia (AML) cells with internal tandem duplications in fms‐like tyrosine kinase 3 (FLT3), referred to as FTL3‐ITD, with proteasome inhibitor bortezomib triggered autophagy‐dependent degradation of FLT3‐ITD and improved the overall survival in a xenografts.^104^ Further, the proto‐oncoprotein AF1Q, which is often overexpressed in AML and myelodysplastic syndrome and associated with an unfavorable prognosis, was targeted for breakdown by CMA.^105^, ^106^ Thus, autophagy and specifically CMA can clear various (proto)oncoproteins, and repression of this type of autophagy might contribute to tumorigenesis. Of note, autophagy can also aid the breakdown of tumor suppressor genes, like p53, as will be described below.

### 3.2 Autophagy in cancer maintenance

As evident from the preceding sections, autophagy can have a tumor suppressor function and is often downregulated in cancer. However, there is also clear evidence to suggest that autophagy is required for cancer (stem) cell maintenance. Indeed, increased autophagic flux or increased dependency on functional autophagy have been reported for various types of cancer, such as melanoma, CML, AML, and RAS‐driven cancers.^107^, ^108^, ^109^, ^110^, ^111^ For example, in solid cancers such as breast cancer and melanoma, increased LC3 puncta positively correlated with a more aggressive phenotype.^110^ Further, autophagic flux can aid cancer cell survival during cellular stress conditions, such as hypoxia and starvation.^67^, ^112^, ^113^ In addition, changes in autophagy can contribute to the maintenance of so‐called CSCs, a self‐renewing subpopulation of cancer cells with stem cell properties that for certain types of cancer, such as AML, is thought to drive the disease. The various roles of autophagy in cancer maintenance are detailed below (illustrated in Figure 3B).

#### 3.2.1 Autophagy in the maintenance of CSCs function

CSCs are characterized by elevated levels of autophagy compared to more differentiated cancer cell populations, an observation confirmed in multiple cancer types, including urinary bladder and breast cancer.^108^, ^114^, ^115^ These CSCs expressed high levels of essential autophagy genes to maintain CSC properties and to remain dormant.^114^, ^116^ Further, elevated autophagy was required for the CSC‐mediated development of tumors in vivo in leukemia and breast cancer.^115^, ^117^, ^118^ However, the differentiation‐dependent level of autophagy is not specifically linked to malignantly transformed cells. Also normal hematopoietic, mesenchymal, and skin stem cells, have a higher level of autophagy as compared with more differentiated cells.^119^, ^120^ Thus, primitive cells have high autophagy levels in association with low ROS levels, which might be a protective mechanism for maintaining stem cell properties.^119^, ^120^ Correspondingly, the function of normal HSCs was lost in ATG7 and ATG12‐knockout mice. In the long term, this loss of function did coincide with the development of myeloproliferative syndrome, possibly a consequence of defective mitochondrial clearance in association with high ROS levels.^82^, ^118^, ^121^ Also, deletion of ATG5 or ATG7 in a mixed lineage leukemia murine AML model affected the survival and was associated with a decrease in a number of functional CSCs and a strong decrease in leukemic blasts in the peripheral blood indicating that autophagy has a critical function in leukemia maintenance.^118^ Similar findings were obtained with a bladder cancer cell line, and with breast cancer mammospheres, a model of CSCs with high levels of Beclin‐1 and an increase in autophagy.^114^ Thus, autophagy seems to be essential to preserve CSC function and to increase survivability.

#### 3.2.2 Oncogenic mutations and autophagy

In established cancers, several oncogenes have been shown to induce autophagy and, thereby, contribute to cancer maintenance. For instance, oncogenic FLT3–ITD–positive AMLs cells are characterized by high levels of autophagy.^122^ Both pharmacological as well as genetic inhibition of autophagy in FLT3‐ITD in human AML cells markedly reduced cell proliferation and overcame acquired resistance to FLT3 inhibitors in mice. In addition, cancer driven by certain oncogenic RAS mutations as observed in a broad spectrum of tumors including colon, lung, and pancreatic cancers, appears to heavily depend on functional autophagy. For instance, basal levels of autophagy were increased in RAS‐transformed cancer cells even under nutrient‐rich conditions.^112^ Moreover, basal autophagy was strongly increased after overexpression of both mutant HRAS and KRAS in human mammary epithelial cells.^123^ The underlying mechanistic reason for mutant HRAS was found to be the activation of Beclin‐1 interacting partner NOXA, thereby upregulating autophagy.^124^ Genetic inhibition of autophagy in cells overexpressing mutant RAS attenuated glycolysis and inhibited proliferation.^123^ Similarly, ATG7 knockout in KRAS‐driven lung cancer cells increased ROS levels and triggered a striking depletion of the cellular nucleotide pool, which was rescued by supplementation with glutamine.^125^ In mouse models, the knockdown of ATG5 or ATG7 cells in RAS overexpressing cells triggered accumulation of dysfunctional mitochondria and reduced tumor growth.^109^, ^126^ Thus, RAS‐driven cancer cells exploit high levels of autophagy, which may position such cancers as targets for autophagy inhibition.

Further, oncogenic mutations in the tumor suppressor protein p53, a protein best known for its proapoptotic effect upon cellular stress, also clearly affect the autophagy pathway. For instance, elevated levels of autophagy were identified in mutant p53 expressing AML cells, whereas a reverse reduction in autophagy was detected in pancreas and breast cancer cell lines that expressed mutant p53.^108^, ^127^ These apparent contradictory data may be explained by the localization of p53, since p53 mutants that localized to the cytosol repressed autophagy, whereas p53 mutants localized to the nucleus did not.^128^ These clear differences in the effect of p53 mutants on autophagy may also impact on therapeutic response toward autophagy inhibition. Indeed, overexpression of mutant p53 in AML cells reduced the sensitivity toward HCQ treatment.^108^ Analogously, mutated p53 glioblastoma cells were less sensitive for CQ treatment.^129^ In contrast, CQ treatment impaired tumorigenesis in mutant KRAS pancreatic tumors with wildtype p53, but augmented tumorigenesis in the absence of p53.^130^ In this respect, it is important to note that wildtype p53 can differentially affect autophagy, with on the one hand inhibition of autophagy upon binding to proteins involved in autophagosome formation.^128^, ^131^, ^132^, ^133^ On the contrary, wildtype p53 can promote autophagy by inhibiting mTOR or by phosphorylation of Beclin‐1.^134^, ^135^, ^136^ Interestingly, the level of p53 itself is also regulated by autophagy. For instance, wildtype p53 is depleted via autophagy‐mediated degradation in renal cell carcinoma, which allows escaping from apoptotic cell death.^137^ In contrast, suppression of macroautophagy promotes the degradation of mutant p53 via CMA, which sensitizes various human cancer cell lines for cell death.^138^ Further, a truncated p53 isoform that inhibits wildtype p53 is degraded via autophagy.^139^

Thus, various known important oncogenic mutated proteins that are important in cancer maintenance are able to regulate autophagy, in most cases triggering elevated levels of autophagy that may aid in cancer cell survival.

#### 3.2.3 Autophagy in cancer metabolism

Autophagy is a catabolic process whereby redundant organelles and proteins can re‐enter various metabolic pathways. Cancer cells typically metabolize glucose to lactate, even when sufficient oxygen is present to support oxidative phosphorylation, a phenomenon known as the Warburg effect.^140^ Of note, pyruvate kinase (PKM2) is the final enzyme in the glycolytic pathway that controls the glycolytic flux and is therefore important for preventing accumulation of glycolytic intermediates.^141^, ^142^ In cancer, PKM2 breakdown via CMA is increased, whereby reduced PKM2 associates with an accumulation of glycolytic intermediates that are rerouted toward branching biosynthetic pathways to support cancer growth.^143^ Likewise, the rate‐limiting enzyme hexokinase 2 (HK2) of the glycolytic pathway, was found to be selectively broken down via autophagy in liver cancer.^144^, ^145^ Together, this indicates that autophagy can control glycolysis at different levels and thus impacts on cancer metabolism. Indeed, glycolysis in MLL–ENL–driven leukemia is augmented by inhibition of autophagy, although the underlying mechanism remains to be determined.^146^ Of note, enhanced lactate secretion due to the Warburg effect can change the extracellular microenvironmental pH, which in turn can activate autophagy.^147^ For example, in breast carcinoma cells acute acidification led to an increase in LC3 puncta together with an increase in the expression of ATG5 and BNIP3.^148^ Thus, degradation of essential metabolic enzymes by autophagy may impact many aspects of central metabolism in cancer. Corroborating evidence hereof was obtained by labeling of wildtype or ATG7^−/−^KRAS driven lung cancer cells with heavy carbon and nitrogen isotopes, which in the autophagy‐deficient cells identified a significant depletion of amino acids linked to the tricarboxylic acid (TCA) cycle.^149^ Therefore, autophagy may provide cancer cells with a mechanism to efficiently redistribute metabolites enabling metabolic rewiring, which is required for malignant transformation.

#### 3.2.4 Autophagy is upregulated in hypoxic tumor regions

Autophagy is also an important regulatory pathway during adaptation of cancer cells to hypoxic stress occurring in poorly oxygenated regions of the bone marrow due to AML infiltration or in hypoxic regions of solid cancers. Indeed, in xenograft models of human head and neck cancer, autophagy was associated with hypoxic tumor regions.^113^ Under hypoxic conditions, stabilization of hypoxia‐inducible factor 1α (HIF1α) was detected, leading to enhanced levels of Beclin‐1, increased LC3‐II/LC3‐I ratio and degradation of p62, eg, upon treatment of lung cancer cell lines with cisplatin.^150^ Likewise, in adenoid cystic carcinoma, the hypoxia mimetic CoCl_2_ stabilized HIF1α and induced autophagy.^151^ HIF1α activity among others upregulates expression of BNIP3 and BNIP3L, which can activate autophagy by shifting the balance of the regulatory Beclin‐1 hub toward autophagy induction (Figure 2B).^48^, ^151^, ^152^ In glioblastomas, increased expression of BNIP3 or ATG9A contributed to hypoxia‐associated growth, which could be blocked in vivo by HCQ.^153^, ^154^ Importantly, tumor cells in hypoxic regions proved to be particularly sensitive to HCQ treatment.^113^ In a panel of cancer cell lines, hypoxia‐induced cell death increased upon knockdown of Beclin‐1 or ATG7, with autophagy‐deficient cancer cells proliferating less in mouse xenograft models.^155^ Of note, xenografts of wildtype cell lines were characterized by increased LC3 and reduced p62 levels in hypoxic tumor regions, reflecting activation and execution of autophagy.^155^ Therefore, in a broad spectrum of cancers induction of autophagy contributes to survival in poorly oxygenated tumor areas.

#### 3.2.5 Autophagy in anoikis and metastasis

Most cancer patients succumb to their disease due to metastatic spread of the original primary tumor, an event that can occur many years after initial seemingly successful treatment of the primary tumor. During metastatic spread, autophagy is thought to be crucial for cancer cell survival. First, cancer cells that spread to distal organs have to resist cell death due to loss of contact with the extracellular matrix (ECM), termed anoikis. Cells can resist anoikis partly through activation of autophagy as shown for metastatic hepatocellular carcinoma.^156^, ^157^ Similarly, transformed fibroblasts were characterized by a strong increase in autophagy after the loss of ECM contact. Further, anoikis was triggered upon inhibition of autophagy in cancer cell lines driven by either RAS or PI3K.^123^, ^158^, ^159^, ^160^ In an attachment‐free culture model system, tumor spheroids of various cancer cell lines depended on BNIP3‐associated autophagy for survival.^161^ Further, rapamycin‐mediated activation of autophagy improved spheroid growth, while autophagy inhibition induced apoptosis.^161^ Correspondingly, the levels of LC3B were significantly higher in metastases compared with primary tumors in breast cancer, liver cancer, and melanoma.^110^, ^157^, ^162^ Moreover, the incidence of metastases was reduced in metastatic liver cancer cells upon knockdown of Beclin‐1 or ATG5 in a mouse model, due to loss of resistance to anoikis.^157^ Thus, metastatic cells appear to be more dependent on functional autophagy to allow survival in the absence of ECM contact after which metastatic cells remain characterized by higher autophagy levels.

### 3.3 The role of autophagy in cytotoxic cancer therapy

Treatment of cancer cells with cytotoxic drugs inevitably leads to cellular stress. Consequently, activation of autophagy is widely described although, as detailed below, the impact of autophagy on cytotoxic therapy can differ depending on the type of cell death. Moreover, although the underlying cause of intrinsic and/or acquired drug resistance is likely multifactorial and often remains enigmatic, autophagy is increasingly recognized as being an important contributor to therapy resistance. In the sections below, the role of autophagy in cytotoxic cell death will be detailed, after which the role of autophagy in resistance to therapy is discussed.

#### 3.3.1 Autophagy has a distinct impact depending on the type of cytotoxic cell death

Autophagy can be a stress response of cancer cells that enables cells to evade apoptotic elimination. An example hereof is the treatment of a triple‐negative breast cancer cell line with a plant‐derived anticancer drug that induced apoptosis and activated autophagy. Here, inhibition of autophagy with 3‐MA served to augment the level of apoptotic cell death.^163^ Similarly, in colorectal cancer cell lines, a proapoptotic polyamine analog simultaneously induced apoptosis and autophagy, with 3‐MA cotreatment enhancing induction of apoptotic cell death.^164^ In another breast cancer cell line model, the novel therapeutic drug NBT was found to induce autophagy and apoptosis, with apoptosis induction being increased upon CQ treatment.^165^ In CML cell lines, the antitumor agent asparaginase‐induced apoptosis and autophagy.^166^ Blockade of autophagy with three different autophagy inhibitors enhanced asparaginase‐induced cell death. Further, inhibition of autophagy in HeLa cells upregulated expression of p53 upregulated modulator of apoptosis (PUMA) via FOXO3a, which upon cotreatment with etoposide or doxorubicin upregulated apoptosis as defined by enhanced activation of effector caspase‐3/7.^167^, ^168^ This sensitizing effect of autophagy inhibition was abolished in cells lacking PUMA, indicating that FOXO3a‐dependent mechanism induction of PUMA contributes to drug resistance.^167^ Interestingly, an important regulator of initiator caspase‐8 activation, the antiapoptotic protein FLIP, also can regulate autophagy activity by competitive binding to ATG3 and preventing lipidation of LC3.^169^

Reversely, autophagy as part of ACD is required for cytotoxic cell death. An example hereof is cell death induced by a cardiac glycoside in non–small‐lung cancer cell lines, which was characterized by an increase in autophagic flux and was inhibited by 3‐MA.^170^ Treatment with this glycoside was accompanied by activation of the JNK signaling pathway, leading to a decrease in the level of Bcl‐2 and a concomitant shift toward Beclin‐1‐mediated induction of autophagy.^170^ Of note, although glycoside treatment elevated the level of intracellular ROS, antioxidant cotreatment did not prevent glycoside‐induced cell death indicating that ROS is a by‐product of ACD in this setting. In contrast, ROS was causal for ACD induction in triple‐negative breast cancer cells by the compound physagulide P purified from Chinese herbal medicine, with cotreatment with a ROS scavenger inhibiting ACD.^28^ Several other pathways can also be involved in therapy‐induced ACD. For instance, radiation treatment of breast cancer cell lines triggered ACD via activation of p53 and downstream p53 effector protein DRAM.^25^ In this case, cell viability was partially rescued upon treatment with 3‐MA or by knockdown of ATG5 or Beclin‐1.^25^ Further, treatment of breast cancer cells with a so‐called selective estrogen receptor modulator induced ACD via reducing ATP levels.^26^ Conversely, the addition of ATP restored cell viability, coinciding with a reduction in the LC3‐II/LC3‐I ratio, which indicates that ACD was averted.^26^ Furthermore, treatment with the glycan‐binding protein, Galectin‐9, triggered cell death in colon cancer cells, which was blocked by knockdown of Beclin‐1 or ATG5.^171^

In conclusion, autophagy during cytotoxic therapy can either be protective or can be instrumental for cell death induced by certain therapeutics. Thus, depending on the type of drug used in the treatment of cancer, the combination with autophagy inhibitors may be warranted or should be avoided.

#### 3.3.2 The role of autophagy signaling in resistance to cancer therapy

As described above, autophagy during treatment may reduce sensitivity to cytotoxic therapy. Correspondingly, resistance to various types of therapy is characterized by enhanced basal levels of autophagy, as defined by increased conversion of LC3‐I to LC3‐II, increased numbers of LC3B puncta per cell, upregulated numbers of autophagolysosomes, and degradation of p62.^172^, ^173^, ^174^ For example, cisplatin‐resistant clones of ovarian cancer cell lines as well as an oral squamous cell carcinoma cell line were characterized by enhanced levels of autophagic flux.^175^ In radiotherapy‐resistant breast cancer cells, ionizing radiation also elevated basal autophagy levels, indicating a protective effect of autophagy against treatment.^176^ Similarly, treatment of pancreatic cancer, colorectal cancer, and AML cell lines with bortezomib was accompanied by elevated autophagic flux.^172^, ^173^ Importantly, in various cell lines and with different types of drugs, the cotreatment with autophagy inhibitors CQ or HCQ re‐sensitized cells to treatment.^177^, ^178^, ^179^, ^180^ For instance, in breast and esophageal squamous cancer cell lines, chemotherapy or radiotherapy induced an autophagy response accompanied by therapy resistance.^180^, ^181^ The cotreatment with CQ did not only reduce clonogenic survival of malignant cells in vitro, but also reduced tumor burden in murine models.^180^, ^181^ Of note, overexpression of multidrug resistance pumps, such as ABCG2, not only facilitates drug resistance by increasing drug efflux but also by increasing autophagic flux.^182^ In line with this, ABCG2‐mediated drug resistance was strongly inhibited by knockdown of either ATG5 or ATG7.^182^ In this respect, CSCs are also known to overexpress ABC transporters, which may upregulate autophagy and contribute to CSC resistance to chemotherapy.^183^ Further, in CSCs, autophagy was upregulated upon treatment with chemotherapy or photodynamic therapy, which contributed to CSCs survival and promoted therapy resistance.^184^, ^185^ Similarly, AML leukemic stem cells (LSCs) were characterized by elevated autophagic flux upon treatment with BET inhibitors, which contributed to resistance to therapy^186^ (Figure 3C). Of note, since both normal HSCs as well as LSCs need a certain amount of autophagy to survive, there is only a relatively small therapeutic window of autophagy inhibition with HCQ (Figure 3D).

Resistance toward antibody‐based therapy can also be regulated by autophagy, which has mainly been studied for cetuximab, an EGFR‐blocking antibody. For instance, cetuximab‐induced autophagy in various EGFR‐expressing cancer cell lines by downregulation of HIF1α and Bcl‐2, which promoted the association of Beclin‐1 with VPS34^187^ and dose dependently activated Beclin‐1‐mediated autophagy in colon carcinoma cell lines.^188^ Analogously, EGFR tyrosine kinase inhibitors activated autophagy by promoting Beclin‐1‐VPS34 complex formation.^189^ Importantly, chemical inhibition of autophagy or knockout of Beclin‐1 sensitized cancer cells for cetuximab‐induced apoptosis.^187^, ^188^ Interestingly, inactive EGFR is required for the induction of starvation‐induced autophagy.^190^ Together, this data clearly indicates that enhanced autophagy can associate with resistance to various types of cancer therapy. Thus, it is of clear relevance to gain insight into how autophagy facilitates resistance to therapy. In the following sections, the role of key autophagy‐regulating signaling pathways and cancer‐associated genetic mutations will be discussed in the context of resistance to therapy.

#### 3.3.3 Key signaling pathways associated with autophagy‐dependent drug resistance

Many studies have focused on unraveling the mechanisms by which chemotherapy and radiation therapy induce resistance, with several key upstream signaling components being implicated. Most notably, deregulation of the upstream autophagy regulatory system AMPK, which can both activate ULK1 and repress mTOR signaling to promote autophagy, has been reported. For instance, treatment of a colorectal cancer cell line with the drug salidroside activated protective autophagy alone as well as in combination with other antitumor agents via activation of AMPK.^191^ When AMPK activity was blocked using a kinase inhibitor, autophagy was reduced as evidenced by a decrease in LC3‐II/LC3‐I ratio, which synergistically enhanced the cytotoxic effects of combined salidroside and chemotherapy treatment.^191^ In other studies, upregulation of autophagy was attributed to direct activation of ULK1. Specifically, AML LSCs that were resistant to treatment with BET inhibitor in vitro were characterized by ULK1 activation.^186^ In contrast, no ULK1 activation was detected in cells sensitive to BET inhibitor treatment. Interestingly, although ULK1 is supposed to be downstream of AMPK signaling, AMPK phosphorylation was detected in both BET inhibitor–sensitive and –resistant cells. Thus, resistance to treatment in these LSC appears to stem from ULK1 signaling that increases autophagic flux.^186^ In a follow‐up study, pharmacological inhibition of AMPK did induce apoptosis in BET‐resistant LSCs. AMPK and ULK1 were found to have a similar cytoprotective mechanism against chemotherapeutics in primary pancreatic cancer cells as well as pancreatic cell lines.^192^ Further, in a t(8;21) AML model, Kasumi‐1 cells survived short‐term treatment with histone deacetylase inhibitors by upregulation of autophagy.^193^ However, interactions between AMPK and mTOR were not investigated and long‐term resistance was not examined. Resistance to therapy due to upregulated autophagy can also be acquired through repression of the mTOR pathway as demonstrated for dexamethasone treatment in various leukemic cell lines.^194^ Similarly, activation of autophagy in an imatinib‐resistant CML line and in cisplatin‐resistant lung carcinoma cells was due to repression of mTOR signaling.^195^, ^196^ Altered signaling of upstream regulators of mTOR caused this repression of mTOR signaling, eg an increase in phosphorylation/activation of AMPK or a decrease in Akt signaling.^194^, ^196^ In targeted therapy, mTOR inhibitors as single agents did induce autophagy, but were ineffective anticancer therapeutics.^197^ However, when mTOR inhibitors were combined with autophagy inhibitors, prominent antileukemic effects were detected.^197^ In clonogenic assays, primary AML cells formed fewer colonies in combination therapy than single treatment. Similarly, knockdown of ULK1 in combination with mTOR inhibitor reduced the colony‐forming potential of primitive AML precursors.^197^

Another pathway involved in autophagy‐mediated resistance to therapy is the MAPK pathway, with chemotherapeutic treatment of hepatocellular carcinoma cell lines leading to increased MEK and ERK activity and induction of cytoprotective autophagy.^198^ This induction of autophagy was partly blocked by MEK inhibition.^198^ In cell lines carrying the oncogenic BRAF V600E mutation that have aberrant constitutive MAPK signaling, treatment with the specific V600E inhibitor vemurafenib resulted in AMPK–ULK1‐mediated autophagosome accumulation.^199^ Autophagy was similarly upregulated in BRAF‐mutated primary melanoma samples treated with BRAF inhibitor compared with baseline untreated samples. Interestingly, here induction of autophagy did not occur through AMPK‐ULK1 signaling, but was likely attributable to induction of endoplasmic reticulum (ER) stress response through CHOP, ATF4, and eIF2α.^200^ Similarly, in cutaneous BRAF‐mutated melanoma cell lines enhanced basal autophagy was observed.^201^ Oncogenic BRAF led to chronic ER‐stress, which in turn activated the JNK‐signaling cascade and contributed to autophagy induction, leading to therapy resistance.^201^ Of note, combined treatment of vemurafenib with autophagy inhibitor CQ almost completely blocked tumor growth in a xenograft mouse melanoma model, highlighting that cytoprotective autophagy was at least partially associated with resistance to vemurafenib. Thus, various types of chemotherapy as well as targeted drugs can trigger activation of autophagy that contributes to resistance to therapy.

#### 3.3.4 HMGB1 positively regulates autophagy, contributing to therapy resistance

Recent evidence suggests that the nuclear protein HMGB1 is another critical regulator of autophagy that can mediate resistance during cancer treatment. Although normally in the nucleus, HMGB1 can translocate to the cytoplasm upon stress where it directly interacts with Beclin‐1 and displaces Bcl‐2. Consequently, cytoplasmic HMGB1 can activate autophagy. Many studies have linked increased HMGB1 protein levels to autophagy and therapy resistance.^202^, ^203^, ^204^, ^205^ For instance, upregulation of HMGB1 occurred during cisplatin treatment in non–small‐cell lung cancer cell lines, which associated with enhanced autophagy.^206^ Knockdown of HMGB1 reduced the levels of autophagy and increased cell death, with knockdown of HMGB1 being more efficient than treatment with well‐known autophagy inhibitor 3‐MA.^206^ Similarly, treatment with docetaxel upregulated HMGB1 protein, leading to enhanced autophagy levels.^207^ Upon continuous treatment with docetaxel, cells became resistant to therapy, with sensitivity being restored by knockdown of HMGB1 and reducing tumor growth in a xenograft model.^207^ In an analogous fashion, treatment of leukemic cell lines with different chemotherapeutic drugs upregulated expression of HMGB1. Upregulation of HMGB1 was associated with enhanced LC3‐II/LC3‐I ratios and protected from treatment‐induced cell death, which was prevented by knockdown of HMGB1.^208^ HMGB1 mediated resistance to chemotherapy via mTOR and Beclin‐1 was further reported in several different cancer cell lines.^204^, ^207^, ^208^ As discussed above, various other factors can induce mTOR, thereby, facilitating resistance to chemotherapy mediated by autophagy.

#### 3.3.5 MicroRNAs in autophagy during treatment resistance

Several lines of evidence have emerged that indicate that microRNAs (miRNA), small noncoding RNAs that degrade mRNA and thereby reduce translation, may also play a regulatory role in autophagy signaling in therapy resistance. For instance, the reduced expression of miR‐23b in radiotherapy‐resistant pancreatic cancer cell lines enhanced the level of autophagy when compared with radiosensitive cell lines.^209^ MiR‐23b directly targeted and reduced ATG12 expression and overexpression of this miRNA in radiotherapy‐resistant cells blocked autophagy, as evidenced by reduced LC3‐II/LC3‐I ratio and reduced numbers of autophagosomes per cell, and re‐sensitized cells to radiation treatment.^209^ In epithelial ovarian cancer cell lines that were resistant to cisplatin treatment, a similar decrease in the level of miR‐429 was detected, which was associated with enhanced levels of autophagy.^210^ Correspondingly, overexpression of miR‐429 reduced autophagy via downregulation of ATG7 and increased cellular sensitivity to cisplatin treatment. Furthermore, doxycycline treatment reduced the expression of miR‐30a, a microRNA that directly targets Beclin‐1 mRNA, whereas the levels of miR140‐5p that targets IP3k2 mRNA were increased.^211^, ^212^ In both cases, induction of autophagy was enhanced and contributed to therapy resistance. In addition, treatment of colorectal cancer cells with cetuximab was associated with downregulation of another Beclin‐1 mRNA‐targeting miRNA, miR‐216b, again yielding elevated activation of autophagy and resistance to therapy.^188^

In conclusion, although still in early stages the available data collectively suggest that downregulation of various miRNAs can directly activate cytoprotective autophagy during therapy by upregulation of key components of the autophagy machinery. Thus, reduced miRNA expression appears to be causally related to autophagy‐mediated resistance to therapy.

#### 3.3.6 Hypoxia as autophagy activating signal in therapy resistance

Several studies highlight that hypoxia‐induced autophagy contributes to resistance to therapy. For instance, in primary glioblastoma tissue samples, administration of the vascular endothelial growth factor neutralizing antibody bevacizumab increased tumor hypoxia. In turn, this hypoxia associated with upregulation of cytoprotective autophagy.^154^ Correspondingly, autophagy inhibition upon bevacizumab treatment of xenografts derived from glioblastoma multiforme patients resulted in increased survival.^153^ In a study conducted on breast cancer cell lines, hypoxia itself did not induce autophagy. However, upon taxol treatment in hypoxic conditions cancer cells did appear to activate cytoprotective autophagy through inhibition of the mTOR pathway.^213^ Similarly, chemotherapy resistance in triple‐negative breast CSCs was attributed to a combination of hypoxia and upregulation of autophagy occurring in xenograft models from these patients.^214^ Overall, this data implies that hypoxia‐mediated resistance to therapy is at least partly due to the induction of cytoprotective autophagy.

## 4 PART II: THE ROLE OF AUTOPHAGY IN THE TUMOR MICROENVIRONMENT

The tumor microenvironment is a specialized niche created during tumor development that plays an important role in terms of cancer progression, survival, and response to therapy. This microenvironment comprises of many different cell types, including fibroblasts, mesenchymal stem cells (MSCs), endothelial cells, and immune cells. All of these cell types to a different extent use autophagy in cellular functioning in cancer, with, eg, autophagy in stromal cells such as fibroblasts promoting tumorigenesis, whereas autophagy in immune cells such as cytotoxic T‐cell (CTLs) facilitates execution of anticancer immune responses. Thus, cells within the microenvironment may have opposing requirements for autophagy that may prove difficult to reconcile for autophagy‐targeting therapy in cancer. In this section, we will attempt to capture the role and importance of autophagy and the impact of potential therapeutic targeting of autophagy for several crucial tumor microenvironmental constituents, namely cancer‐associated fibroblasts (CAFs) and MSCs, endothelial cells, innate and adaptive immune cells.

### 4.1 Autophagy in the tumor microenvironment; stromal cells

#### 4.1.1 Autophagy in stromal cells promotes cancer cell growth and survival

A positive influence of fibroblasts on cancer cell growth is well documented, with, eg, enhanced growth rates for both fibroblasts and colon cancer cell lines in cocultures, as well as enhanced growth rates of head and neck squamous cell carcinoma (HNSCC) cells and breast carcinoma cells.^215^, ^216^, ^217^ Similarly, primary patient‐derived AML cells survive and proliferate better in coculture with mouse stromal cells or human MSCs.^218^, ^219^, ^220^ In cocultures, fibroblasts were characterized by elevated levels of autophagy as, eg, evidenced by the accumulation of LC3‐positive vesicles.^215^, ^216^, ^217^ Importantly, inhibition of autophagy markedly attenuated the beneficial impact of fibroblast in such cocultures. Specifically, inhibition of autophagy using 3‐MA treatment reduced the growth rate of colon cancer cells, whereas treatment with CQ or knockdown of Beclin‐1 in fibroblasts prevented the increase in HNSCC proliferation in cocultures. Together these data indicate that cancer cells induce and exploit elevated levels of autophagy in stromal cells for their aberrant growth. In this respect, fibroblasts isolated from tumors indeed had higher autophagy activity than normal fibroblasts.^216^

In addition to promoting cancer cell proliferation, there are some clues that autophagy in stromal cells also helps to promote cancer cell survival and can protect against anticancer therapy. Specifically, in cocultures of cancer cells with fibroblasts the basal level of apoptosis in cancer cells decreased, a phenomenon reversed by inhibition of autophagy using CQ.^217^, ^222^, ^223^ Of note, this effect on basal apoptosis was significant, yet small with the basal level of apoptosis dropping from 5% in breast cancer monocultures to 1% in fibroblast cocultures. More importantly, fibroblasts protected breast cancer cells against treatment with tamoxifen, yielding 85% apoptosis in monocultures versus 45% in fibroblast cocultures.^222^ However, the relative importance of autophagy in this setting remains to be determined, as no autophagy inhibitors were applied to identify the impact of autophagy. Similarly, under serum deprivation conditions, MSCs were able to limit the induction of apoptosis in lung cancer cell lines through activation of autophagy.^224^ Interestingly, CAFs also resist stress better than normal fibroblasts, as fibroblasts isolated from ovarian cancer patients were more resistant to oxidative stress, with sensitivity being restored by Beclin‐1 or ATG5 knockout.^221^ Thus, autophagic signaling in stromal fibroblasts and MSCs can contribute to survival and growth of cancer cells.

#### 4.1.2 Soluble factors secreted in stromal cell/cancer cocultures affect autophagic signaling

In many cases, the positive effect of fibroblasts on cancer cell growth was retained when cells were cultured in the absence of direct cell‐cell contact or when the conditioned medium of fibroblasts was used.^215^, ^216^, ^225^ In the latter case, the conditioned medium of CAFs outperformed that of normal fibroblasts.^216^, ^225^ Further, the supernatant of CAFs also protected melanoma and lung cancer cells from radiation‐induced cell death.^226^ This protumorigenic effect of secreted factors was due to autophagy signaling, as conditioned medium from CAFs pretreated with CQ failed to promote proliferation, migration, and invasion.^216^ Thus, CAFs secrete soluble factors through autophagy (called “secretory autophagy”) that are beneficial for cancer cells. Several secreted factors were identified, including various cytokines such as IGF1, IGF2, and CXCL12, all of which promoted survival of A375M melanoma and A549 lung cancer cells after radiation.^226^ Further, injection of CAFs at the site of tumors previously eradicated by radiation accelerated the subsequent development of tumor recurrence, which was abrogated by IGF2 knockout or 3‐MA treatment.^226^ This finding highlights the importance of this cytokine produced by CAFs under autophagy for cancer cell survival. Importantly, IGF2 produced by CAFs also induced autophagy in cancer cells, indicating a feed‐forward loop to promote autophagy in the tumor microenvironment. In a similar fashion, IL‐6 and IL‐8 secretion by CAFs was reduced upon knockdown of Beclin‐1, which decreased migration of HNSCC cells.^216^ Of note, direct addition of IL‐6 and IL‐8 to HNSCC cells promoted migration to a similar extent as coculture with CAFs, highlighting the importance of those cytokines for the autophagy‐mediated effect of fibroblasts. Cytokine production by fibroblasts was attributed to bFGF‐induced autophagy, with knockdown of bFGF in HNSCC cells reducing autophagy in fibroblast and reducing cytokine secretion. Similarly, TGF‐β secreted by breast cancer cells was shown to induce autophagy in CAFs.^227^ Thus, factors secreted by cancer cells can trigger activation of autophagy in CAFs, which concomitantly results in secretion of cytokines that elevate autophagy and have a protumorigenic effect on cancer cells. Hence, inhibiting autophagy in both cancer cells and cancer‐associated stromal cells likely outperforms inhibiting autophagy in cancer cells only. Indeed, simultaneous knockout of ATG7 in both MSCs and AML cells increased the sensitivity to cytarabine treatment compared with ATG7 knockout in AML cells alone.^228^

#### 4.1.3 Cancer cells trigger metabolic reprogramming of cancer‐associated fibroblasts

In coculture experiments of fibroblasts and cancer cells, hypoxic stress was elevated in the fibroblast population, leading to the induction of autophagy and metabolic reprogramming. For instance, in coculture with breast cancer cells, HIF1α and NFκB signaling activated autophagy and, more specifically, mitophagy in CAFs.^223^ Similarly, coculture of fibroblast and colon cancer cells induced oxidative stress in fibroblasts and elevated the level of autophagy.^215^ Correspondingly, expression of constitutively active HIF1α in fibroblasts also induced autophagy/mitophagy, whereas treatment with HIF1α inhibitor echinomycin reduced levels of autophagy.^229^ Due to elevated mitophagy, the mitochondrial mass in fibroblasts was strongly reduced when cocultured with cancer cells.^217^ This resulted in a metabolic shift from the TCA cycle to the glycolytic pathway, also yielding increased production of ketones and lactate.^215^ A similar shift was detected in fibroblast engineered to overexpress the p53‐inducible autophagy inducer DRAM, leading to elevated autophagy, reduced mitochondrial mass, and an increase in secretion of ketones and lactate.^230^ In line with this, overexpression of ATG16L1 or BNIP3L, to induce autophagy, reduced fibroblast mitochondrial activity and increased glycolytic pathway activity.^231^ Interestingly, lactate and ketones produced by fibroblasts were utilized by cancer cells leading to increasing mitochondrial mass and mitochondrial oxidative metabolism of cancer cells in coculture with fibroblasts.^217^ Of note, HIF1α also directly activates the glycolysis pathway.^232^ Therefore, it is unclear whether elevated autophagy is the cause of glycolysis induction or that both pathways are simultaneously induced upon hypoxic stress. Taken together, cancer cells trigger hypoxic stress in fibroblasts leading to activation of autophagy and mitophagy and a metabolic switch from the TCA cycle to glycolysis. The metabolites produced by these fibroblasts are subsequently consumed by cancer cells and contribute to cancer cell growth and survival.^226^

Autophagy in fibroblasts has further been linked to reduced caveolin‐1 (cav‐1) expression in the stroma of breast cancer patients, a feature associated with poor survival.^233^, ^234^ Specifically, cav‐1 expression was downregulated in fibroblasts which were modulated to have elevated levels of autophagy.^217^, ^223^, ^229^, ^231^, ^235^, ^236^ Correspondingly, cav‐1 expression inversely correlated with autophagy and mitophagy in cell lines and patient‐derived human breast cancer samples.^223^ In mice, coinjection of breast cancer cells with fibroblasts yielded larger primary tumors and an increase in metastases, especially when fibroblasts were modulated for increased autophagic flux and reduced cav‐1 levels.^217^, ^235^, ^236^

Taken together, elevated levels of autophagy in CAFs promote cancer cell growth and survival, which among others is due to a metabolic switch of fibroblasts to glycolysis and the secretion of glycolytic by‐products.

#### 4.1.4 Autophagy in endothelial cells modulates angiogenesis

Fast expanding tumors require sufficient angiogenesis. The importance of autophagy in this process is not yet thoroughly investigated, although some studies show that autophagy influences angiogenesis. For instance, the knockdown for ATG5 or treatment with 3‐MA of bovine aortic endothelial cells blocked angiogenesis (as determined by reduced tube length, migration, branching), whereas ATG5 overexpression promoted angiogenesis.^237^ Similarly, human dermal microvascular endothelial cells were blocked in angiogenesis upon 3‐MA or CQ treatment, whereas rapamycin promoted angiogenesis.^238^ In contrast, in the context of cancer cells, the inoculation of B16F10 melanoma cells in wildtype or heterogeneous Beclin‐1‐knockdown mice showed more angiogenesis in the Beclin‐1‐knockdown mice with concomitant bigger tumors and more lung metastasis.^239^ However, this effect was only seen under hypoxia and not normoxia. Also in vitro, Beclin‐1‐knockdown cells from lung epithelial cells and Beclin‐1 siRNA in wildtype cells yielded more angiogenesis under hypoxia but not under normoxia, which was regulated via HIF2α. Since cancer cells induce a state of hypoxia in adjacent fibroblast (as described above) it can be hypothesized that this also occurs in endothelial cells, leading to a reduction in angiogenesis when endothelial autophagy levels are high. In addition, modulating autophagy per se can suppress angiogenesis as has been shown in a model in chick eggs and human umbilical endothelial cells.^240^ Here, both rapamycin and 3‐MA disturbed normal blood vessel formation. Further, both downregulation of Atg7 or overexpression suppressed tube formation. However, rapamycin promoted endothelial cell migration. Thus, it is also possible that different processes in angiogenesis are differentially regulated by autophagy. Thus, it is currently unclear whether autophagy in endothelial cells should be inhibited or promoted to optimize anticancer effects.

### 4.2 Autophagy in cancer immunity; the cancer cell side of the coin

From the above paragraphs, it is clear that autophagy directly impacts on cancer proliferation and survival and thus is a target for inhibition in cancer cells. However, elevated autophagy levels in cancer cells can also have a diverse impact on anticancer T‐cell immunity. In brief, anticancer T‐cell immunity is a multilayered and intricately regulated process, which pivots on the recognition of antigenic peptides presented on the cancer cell surface in the so‐called major histocompatibility complex (MHC) class I to CTLs. Upon recognition of an MHC/peptide complex, the CTLs form an immunological synapse with the cancer cell and secrete cytotoxic and tumoricidal proteins, such as granzymes and perforins. Consequently, the cancer cell is eliminated by apoptosis.

**Figure 4:**
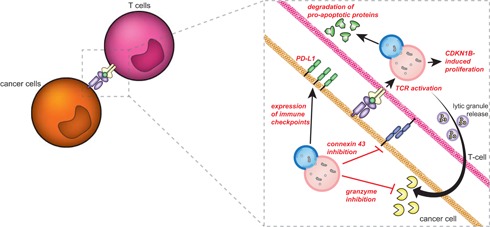
Autophagy in the tumor microenvironment impacts on anticancer immunity. Autophagy in cancer cells inhibits the anticancer immune response by reducing the efficacy of cytotoxic T‐cell and natural killer cell–mediated lysis by degrading granzyme B and connexin‐43. Further, autophagy is also required for T‐cell proliferation, survival, and induction of T‐cell memory by degrading proapoptotic proteins and maintaining mitochondrial homeostasis. Therefore, nonselective inhibition of autophagy in the tumor microenvironment will not only promote anticancer effects at the level of stroma and cancer cells, but will also dampen anticancer immune responses. CDKN1B, cyclin‐dependent kinase inhibitor 1B; PD‐L1, programmed cell death 1 and its ligand; TCR, T‐cell receptor [Color figure can be viewed at wileyonlinelibrary.com]

Autophagy affects many aspects of this immune response and in cancer cells, for instance, inhibits proper formation of the immunological synapse and reduces the cytolytic potential of CTLs and natural killer (NK) cells (Figure 4). In addition, autophagy in cancer cells can have both a proimmune and an immune inhibitory effect by modulating the expression of immune checkpoints and steering the induction of so‐called immunogenic cell death (ICD). In this section of the review, the role of autophagy in anticancer immunity is discussed in detail.

#### 4.2.1 Autophagy in cancer cells inhibits anticancer immunity by reducing the sensitivity toward NK‐ and CTL‐mediated lysis

Cancer cells are lysed when they express MHC‐I molecules containing tumor‐derived antigenic peptides that are recognized by CTLs. To evade recognition and elimination by the immune system, cancer cells therefore often downregulate their MHC‐I expression.^241^, ^242^ Although this is mainly regulated by genetic mutations and epigenetic modifications, MHC‐I molecules are also degraded by autophagy. Indeed, inhibition of autophagy augmented cell surface expression of MHC‐I induced by treatment of melanoma cells with the immunostimulatory cytokine interferon γ (IFNγ).^243^ Importantly, the loss of MHC‐I expression (“missing self”) is also being recognized by NK cells, leading to the elimination of the cancer cell.^241^ However, several reports detail that high levels of autophagy in cancer cells reduces the efficacy of NK and CTL‐mediated cell lysis. For instance, autophagy in cancer cells affects the stability of the immunological synapse generated between the cytolytic immune cell and its target cell. Specifically, the formation of gap junctions, which requires connexin proteins such as connexin‐43, normally facilitates the exchange of small molecules between effector and target cell and is required for NK lysis.^244^ In melanoma cells, the accumulation of connexin‐43 at the immunological synapse was reduced under hypoxia, which was restored by inhibition of autophagy.^245^ Consequently, NK‐mediated cell lysis was restored. Interestingly, the gap junction protein connexin‐43 also transports active granzyme B, one of the main cytotoxic molecules of CTLs and NK cells, into the target cell.^244^ Thus, the degradation of connexin‐43 by autophagy may affect cytolysis in various ways. Interestingly, granzyme B is also a target of autophagy‐mediated breakdown, especially under hypoxic conditions. Correspondingly, granzyme B was predominantly detected in LC3 and Rab5‐positive fractions in hypoxic cells.^246^ Hence, autophagy in cancer cells contributed to resistance to lysis of lung cancer cell lines, breast cancer cells, and melanoma cells in hypoxic conditions by degrading granzyme B.^246^, ^247^, ^248^ A similar effect was detected upon hypoxia‐independent activation of HIFs, as seen in renal cancer with mutations in the von Hippel‐Lindau gene. In these cells, autophagy was upregulated, and cells were resistant toward NK‐mediated cell lysis.^249^ Autophagy not only affects CTL lysis in hypoxia, but also in normoxia with reduced sensitivity of melanoma cells toward CTL‐mediated lysis compared with healthy cells.^250^ In all cases, CTL‐ or NK cell–mediated lysis was restored by inhibition of autophagy.^246^, ^249^, ^250^ Of note, upregulation of autophagy also confers resistance to CTL lysis during the so‐called epithelial‐mesenchymal transition, a step necessary for cancer progression and metastasis, in breast carcinoma cells.^251^ Sensitivity to CTL‐mediated cell lysis in this setting was partly restored by Beclin‐1 knockdown. Thus, autophagy may affect cancer cell sensitivity to immune cell lysis at multiple levels.

Taken together, increased autophagy in cancer cells negatively affects sensitivity toward NK‐ and CTL‐mediated cell lysis through degradation of granzyme B and inhibition of the immunological synapse.

#### 4.2.2 Autophagy in cancer cells regulates the expression of immune checkpoints

Immune checkpoints are coinhibitory receptor/ligand pairs that serve to dampen immune cell activity.^252^ A prominent example hereof is the receptor programmed cell death 1 (PD‐1) and its ligand (PD‐L1), which are expressed on activated T‐cell and antigen‐presenting cells (APCs), respectively. This checkpoint is a crucial inhibitor of anticancer T‐cell responses in the tumor microenvironment.^253^ Interestingly, activation of autophagy using mTOR inhibitor rapamycin decreased the expression of PD‐L1 in lung cancer cells in vitro and in vivo, whereas activation of mTOR increased expression of PD‐L1.^254^ Correspondingly, almost all human lung cancer patient samples (~90%) expressing PD‐L1 were characterized by increased mTOR signaling, whereas the majority (83%) of tumors negative for PD‐L1 also stained negative for active mTOR. Of note, signaling through PD‐L1 itself activated mTOR signaling in melanoma and ovarian cancer cells, with PD‐L1 blockade decreasing mTOR signaling in a mouse model of pancreatic cancer.^255^, ^256^ Thus, PD‐L1 activates mTOR and in a feed‐forward loop upregulates expression of PD‐L1, signaling that proceeded via mTORC1 and not mTORC2.^254^, ^255^ In line with the fact that mTORC1 is a major (negative) regulator of autophagy, melanoma, and ovarian cancer cells with low autophagic flux expressed higher levels of PD‐L1 than cells with high autophagic flux.^255^ Subsequent treatment with rapamycin to induce autophagy triggered a reduction in PD‐L1 expression and reactivated T‐cell–mediated anticancer immunity.^254^ Further, cotreatment with rapamycin and PD‐1 blocking antibodies more effectively reduced tumor growth than single treatment and was accompanied by reduced numbers of regulatory T‐cell and increased CD3^+^ T‐cell numbers. Thus, low levels of autophagy signaling associated with an increase in expression of PD‐L1.

#### 4.2.3 Autophagy in cancer cells modulates the induction of immunogenic cell death

Autophagy can further impact on the process of ICD, a type of apoptosis that stimulates the development of anticancer T‐cell responses. ICD is induced by certain anticancer therapeutic strategies such as anthracyclines like mitoxantrone or doxorubicin, radiation therapy and photodynamic therapy (PDT).^257^ ICD requires the translocation of calreticulin to the cell surface and the release of several immune‐stimulating factors, among which HMGB1 and ATP.^258^, ^259^ During ICD, depletion of ATG5 or ATG7 in CT26 murine colon cancer cells or knockdown of Beclin‐1 reduced ATP‐release upon anthracycline treatment and inhibited in vivo anticancer immunity.^258^, ^260^ In contrast, ATG5 knockdown did not reduce ATP secretion in bladder cancer and melanoma cells after hypericin‐mediated PDT, although in this case knockdown of ATG5 did increase cell surface exposure of calreticulin.^261^ In contrast, calreticulin exposure was not affected upon anthracycline treatment of autophagy‐deficient and ‐competent CT26 cells or mouse embryonic fibroblasts (MEFs).^258^

The impact of autophagy inhibition on ICD also depends on which stage of autophagy is blocked, with calreticulin exposure being strongly reduced by blocking autophagy at early stages by silencing of ULK1, Beclin‐1, or ATG5.^260^ In contrast, calreticulin exposure was increased when autophagy was blocked at a late stage using vincristine, CQ, and bafilomycin A1. Of note, the induction of autophagy alone using rapamycin or mTOR siRNA was not enough to induce ATP secretion or calreticulin exposure.^258^, ^260^ Indeed, activation of the proapoptotic effector caspase‐8 is known to be pivotal for calreticulin exposure during ICD.^262^ Taken together, autophagy modulates the release of ATP and the cell surface exposure of calreticulin and thus contributes to ICD of cancer cells.

### 4.3 Autophagy in cancer immunity: the T‐cell side of the coin

Autophagy not only impacts on the immunogenicity of cancer cells, but is also pivotal for the correct functioning of APCs and T‐cell. For instance, the development of de novo T‐cell responses requires the presentation of antigenic peptides by professional APCs, most notably dendritic cells (DCs). DCs present the antigenic peptides to CTLs in the context of MHC‐I and further present peptides from endocytosed material to CD4^+^ helper T‐cell in the context of MHC class II. Activation of helper T‐cell licenses the DC to stimulate the clonal expansion of CTLs and is of great importance for anticancer T‐cell responses.^263^, ^264^, ^265^ Autophagy is critically involved in antigen presentation in both MHC‐I and MHC‐II in DCs. Finally, autophagy is also important in the functional activity of the immune cell itself, with T‐cell activity and generation of T‐cell memory requiring autophagy. Thus inhibition of autophagy in the context of cancer therapy also likely affects the activity of intratumoral T‐cell. Of note, the investigation of autophagy in the context of cancer immunity is in its infancy, but as detailed below, knowledge of core principles by which autophagy regulates T‐cell functioning has been gained among others in viral infection models.

#### 4.3.1 Autophagy modulates surface MHC expression and alters the presentation of antigenic peptides

**Figure 5:**
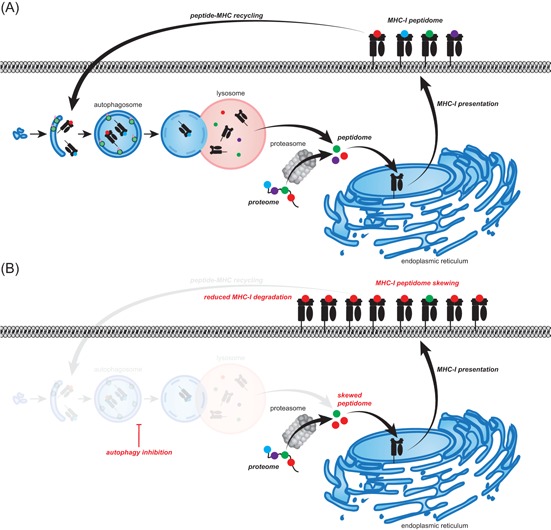
Autophagy contributes to the formation of antigenic peptides in antigen‐presenting cells (APCs). Professional APCs, such as dendritic cells and macrophages, display antigenic peptides in the context of major histocompatibility complex class 1 (MHC‐I) or MHC‐II molecules to T‐cells, which will trigger an immune response. Autophagy reduces MHC‐I surface levels, which is converted upon autophagy inhibition. However, autophagy is also required for the generation of antigenic peptides. The inhibition of autophagy will therefore skew the peptidome, yielding less diversity In the antigens presented to T‐cells. Indeed autophagy, inhibition limits T‐cell activation by APCs [Color figure can be viewed at wileyonlinelibrary.com]

Anticancer T‐cell immunity is induced upon recognition of antigenic tumor peptides presented on the cell surface of professional APCs, such as DCs. However, the surface expression of the MHC‐I/peptide complex is directly affected by autophagy in DCs and macrophages (Figure 5A). For instance, expression of MHC‐I on murine macrophages and DCs was upregulated upon inhibition of autophagy using various chemical inhibitors or downregulation of core autophagy genes.^243^, ^266^ This upregulation was attributed to slower internalization of MHC‐I.^266^ Thus, in the absence of autophagy, MHC‐I molecules appear to be more stably expressed and less degraded.^266^ Correspondingly, DCs from VPS34‐deficient mice expressed more cell surface MHC‐I as well as MHC‐II.^267^ In contrast, surface expression of MHC‐II on macrophages was downregulated upon inhibition of autophagy using 3‐MA.^166^, ^268^

Thus, inhibition of autophagy upregulates surface expression of MHC‐I, although the impact on the surface expression of MHC‐II is less conclusively established. However, with one notable exception in an influenza model, functional studies highlight that despite an increase in surface MHC‐I expression the inhibition of autophagy actually weakens T‐cell responses.^266^ This weakening can likely be attributed to an alteration in the pool of immunogenic peptides presented in MHC, which has been best determined in the context of so‐called cross‐presentation in DCs. Cross‐presentation is a process which enables the loading of MHC‐I on DCs with extracellular antigens, which is important for activation of, eg, CTL responses in melanoma.^269^ DCs with cross‐presentation capacity are characterized by increased levels of autophagy compared with DCs that do not cross‐present, with inhibition of autophagy reducing MHC‐I‐mediated cross‐presentation of ovalbumin (OVA) and human cytomegalovirus (CMV) peptides.^270^, ^271^ Antigen presentation in MHC‐II was similarly altered upon inhibition of autophagy, with the reduced DC‐mediated processing of an immunodominant mycobacterial peptide, reduced presentation of herpes simplex virus (HSV) antigens, and vaccinia virus Ankara antigens.^272^, ^273^, ^274^ Consequently, antigen‐specific T‐cell responses were downregulated. Thus, autophagy inhibition modified the peptide pool presented in MHC and appeared to reduce the presentation of immunodominant epitopes.

In line with this role of autophagy in presenting appropriate antigenic peptides, knockout of the autophagy‐regulator VPS34 in DCs abrogated the induction of B16 melanoma‐specific CTLs in vivo, yielding a significantly higher incidence of lung metastases.^275^ In this setting, cross‐presentation of OVA peptides derived from apoptotic cells was strongly reduced, although VPS34‐deficient DCs did present higher levels of cytoplasmic OVA‐derived antigens.^276^ In addition, mice with autophagy‐deficient DCs failed to efficiently develop protective Th1 cell immunity and hence died faster upon challenge with a lethal dose of HSV.^273^ Further, starvation‐induced autophagy increased loading of intracellular and lysosomal‐derived peptides on MHC‐II molecules in human B‐lymphoblastoid cells.^277^

Taken together, most studies highlight that inhibition of autophagy negatively affects the MHC‐dependent presentation of tumor antigens by APCs and, thereby, negatively affects T‐cell immunity (Figure 5B).

#### 4.3.2 Autophagy is required for T‐cell proliferation, survival, and generation of protective T‐cell memory

Autophagy plays a crucial in normal T‐cell functioning as evident from the severe reduction in basal peripheral T‐cell counts, especially CTL counts, in autophagy‐knockout mouse models.^89^, ^267^, ^277^, ^278^, ^279^, ^280^, ^281^, ^282^, ^283^, ^284^, ^285^, ^286^, ^287^ Naïve resting T‐cell have only minimal numbers of autophagosomes, but T‐cell receptor (TCR)‐mediated activation triggers a strong increase in autophagosome content in activated CD8^+^CD28^+^ CTLs and activated helper T‐cell subsets.^278^, ^288^, ^289^, ^290^, ^291^, ^292^ Induction of autophagy was augmented by T‐cell costimulation with the cytokine IL‐2 or by 4‐1BB signaling, which was accompanied by an increase in lysosomal content and colocalization of lysosomal LAMP staining with autophagosomal LC3 staining.^278^, ^288^, ^290^, ^291^, ^292^, ^293^ Moreover, in activated T‐cell transfected with an LC3‐GFP‐mCherry construct the LC3‐GFP signal was lost, indicative of fusion of autophagosomes with lysosomes. Thus, T‐cell activation increases autophagosome and lysosome content and increases autophagic flux.^289^

The importance of autophagy activation for T‐cell functioning became apparent upon knockout of various core autophagy genes and upstream autophagy regulators in T*cell. In all cases, this resulted in a poor proliferation of T‐cell upon TCR activation, which was not improved by addition of CD28 or IL‐2 costimulation.^89^, ^267^, ^283^, ^284^, ^285^, ^286^, ^294^, ^295^ Indeed, in T‐cell–specific ATG5 or ATG7‐knockout mouse models a strong decline in reactive CTLs was detected in models of lymphocytic choriomeningitis virus (MCMV), influenza virus or mouse CMV.^280^, ^296^ Notably, autophagy‐deficient T‐cell did express equal levels of the activation markers CD69 and CD25 upon TCR‐stimulation, suggesting that downstream TCR signaling does occur.^89^, ^278^, ^283^ Thus, it currently remains unclear whether the defect in the proliferating capacity of autophagy‐deficient T‐cell is on the level of the TCR or more downstream.

In addition to impaired cell proliferation, CTLs from ATG5‐knockout chimeras were ~50% more apoptotic than control T‐cell, and the viability of helper T‐cell from Beclin‐1‐knockout mice was also strongly reduced.^278^, ^284^ Further, spontaneous apoptosis or apoptosis after TCR activation in T‐cell was increased when core autophagy genes and regulators were knocked out.^89^, ^267^, ^280^, ^281^, ^283^, ^285^, ^287^ Indeed, ATG3 and ATG7‐knockout T‐cell contained more active caspase‐9 indicative of elevated levels of apoptosis, with viability being partly restored upon pan‐caspase inhibition.^284^, ^287^ In addition, upon TCR activation, autophagy‐deficient T‐cell secreted less proinflammatory cytokines, that is, IL‐2 and IFN‐γ, which may also negatively impact on their survival.^282^, ^290^ Taken together, inhibition of autophagy at multiple levels has a negative effect on T‐cell.

Importantly, autophagy is also pivotal for the development of T‐cell memory responses, with no development of protective T‐cell immunity in ATG5‐knockout mice upon rechallenge with influenza.^279^ Similarly, ATG7‐knockout mice vaccinated against MCMV failed to generate a T‐cell response upon re‐infection.^280^ Also the inhibition of CMA in T‐cell by knockdown of LAMP‐2A impaired the control of *Listeria monocytogenes* in mice upon rechallenge.^293^ Of note, residual peripheral T‐cell detected in autophagy‐deficient mouse models displayed a CD44^high^CD62^low^ phenotype, a phenotype typically associated with effector and effector memory T‐cell.^89^, ^267^, ^280^, ^287^ Although this finding is in apparent contrast with the impaired induction of T‐cell memory, a similar “memory‐like” phenotype has been reported in lymphopenia in T‐cell–depleted mice.^297^ Similar to autophagy deficiency, such “memory‐like” T‐cell were, in fact, incapable of generating effective T‐cell immune responses. Thus, autophagy is pivotal for normal T‐cell function and, crucially, for the development of memory T‐cell that provide protective immunity. As a result, systemic, nontargeted application of autophagy inhibitors for cancer treatment likely also has a large negative effect on the function and activity of T‐cell, thereby, hampering the induction and/or execution of proper anticancer immune responses.

#### 4.3.3 Autophagy‐dependent degradation of mitochondria and proapoptotic proteins maintain T‐cell homeostasis

The inhibition of autophagy negatively impacts on T‐cell, likely due to deregulated clearance of organelles and proteins, with transcriptional profiling of ATG5 wildtype vs knockout T‐cell among others suggesting a key involvement in mitochondrial homeostasis.^89^ In line with this analysis, knockout of ATG5 or other core autophagy genes was associated with an increase in mitochondrial mass compared to wildtype T‐cell, with a notable exception being Beclin‐1 knockout.^267^, ^283^, ^284^, ^285^, ^286^ Further, mitochondrial mass increased over time in inducible autophagy‐knockout models and was seen only in “aged” T‐cell in the periphery.^89^, ^267^, ^283^, ^287^ In autophagy‐deficient T‐cell, the increase in mitochondrial mass was accompanied by elevated levels of ROS, whereas activation of autophagy using autophagy‐inducer Torin‐1 reduced ROS levels and increased survival.^280^, ^283^, ^285^, ^286^, ^287^, ^295^ Correspondingly, treatment of hypoxic ATG5‐knockout T‐cell with ROS inhibitor *N*‐acetylcysteine restored T‐cell proliferation and prevented cell death.^279^

Autophagy also directly regulated expression of proapoptotic and antiapoptotic proteins in T‐cell. For instance, caspase‐8 and ‐3 protein levels were strongly increased in Beclin‐1‐knockout T‐cell, whereas no increase in mRNA levels was detected.^284^ Further, activation of autophagy using rapamycin triggered colocalization of LC3 and caspase‐3 and was accompanied by downregulation of caspase‐3 levels.^284^ Similarly, the level of several other apoptotic proteins like Bcl‐2, BIM, Bcl‐xl, Bax, cytochrome C, and AIF were increased upon interference with autophagy, although the impact of autophagy inhibition on these proteins varied among studies, possibly related to the timing of measurements, especially in inducible models.^267^, ^281^, ^284^, ^287^

Finally, autophagy also impacts on several key cell cycle regulators in T‐cell. For instance, TCR activation of T‐cell normally induces autophagy‐mediated degradation of cyclin‐dependent kinase (CDK) inhibitor 1B (CDKN1B), an inhibitor of cell cycle progression.^294^ Inhibition of autophagy prevented CDKN1B degradation upon TCR‐stimulation and, thereby, inhibited proliferation. Similarly, autophagy ensures degradation of Bcl‐10, a mediator of TCR‐to‐NF‐κB signaling, as well as Itch and Rcan1, two inhibitors of TCR signaling.^293^, ^298^

Taken together, the autophagy pathway is important for T‐cell survival and proliferation as it retains mitochondrial homeostasis and ensures the degradation of proapoptotic and antiproliferative proteins. An interesting exception to this rule was recently reported for a specific T‐cell subset, the so‐called Th9 T‐cell, which is reported to have potent anticancer immunity.^299^, ^300^ In this case, inhibition of autophagy prevented degradation of PU.1, the master transcription factor for Th9 cells.^301^ Hence, inhibition of autophagy enhanced differentiation of helper T‐cell to Th9 cells.

#### 4.3.4 The role of autophagy in regulatory T‐cell functioning

Regulatory T (Treg) cells are a subpopulation of CD4^+^ T‐cell that inhibit effector T‐cell responses, with increased Treg infiltration in cancer associating with poor survival.^302^, ^303^ Compared to naïve CD4^+^ cells, Tregs contain more autophagosomes and have higher LC3‐II levels indicating the presence of increased levels of autophagy.^304^ Knockout of autophagy genes induced apoptosis of Treg cells and blocked Treg‐mediated suppression of effector T‐cell responses. This subsequently yielded higher percentages of tumor‐infiltrating CTLs and smaller tumors in a mouse model of colon adenocarcinoma.^304^ Further, animals with autophagy‐impaired Tregs were more prone to develop autoimmune diseases and adoptive transfer of Tregs from VPS34‐knockout mice failed to protect against colitis.^267^ Thus, autophagy is important for Treg immunosuppression. Autophagy inhibition may thus alleviate Treg immunosuppressive activity in cancer.

## 5 CONCLUSIONS AND PERSPECTIVES

Autophagy has a multifaceted impact on the cancer microenvironment and is an interesting target for cancer therapy. In established cancers, autophagy acts as a survival mechanism, eg, in conditions of elevated nutrient demand or low oxygen availability. Importantly, cancer cells elevate their autophagic flux during treatment to gain resistance toward (chemo)therapy. In addition, tumors activate autophagy in adjacent stromal cells to benefit from cytokines, growth factors and nutrients secreted upon induction of this pathway. Further, although the autophagy pathway is often repressed during early tumorigenesis, every cell requires a basal level of autophagy. Therefore, emerging cancer cells with downregulated levels of autophagy might be more reliant on remaining autophagy activity. Thus, therapeutic inhibition of autophagy may be well effective in both cancer cells with a high autophagic flux as well as cancer cells with a low autophagic flux.

Interestingly, many types of malignancies have a high prevalence in the aging population, while it has been suggested that a reduction in autophagy may be a contributor to the aging process. In line with this, autophagy was decreased in two‐third of HSCs of aged mice compared to young mice, with aged HSC with higher autophagy activity having better long‐term regenerative potential.^87^ Further, aging of the hematopoietic compartment is associated with myeloid malignancies, with a lineage skewing of HSCs due to upregulation of myeloid‐specific genes and downregulation of lymphoid genes.^305^, ^306^, ^307^, ^308^, ^309^ Intriguingly, similar myeloid skewing was detected upon monoallelic knockout of ATG7 or ATG12 in mice, which associated with the development of myeloproliferative syndrome.^82^, ^87^ Interestingly, treatment with autophagy inducer rapamycin also enhanced influenza‐specific CD8^+^ T‐cell responses in aged vaccinated mice, but not in aged mice with ATG7 knockout.^280^, ^310^, ^311^ Thus, reduced autophagy during ageing may increase susceptibility to tumorigenesis as well as negatively impact on T‐cell immunity. In this respect, activation of autophagy may even be a strategy for rejuvenation, with the treatment of mice with the autophagy inducer rapamycin extending their lifespan.^312^

An important process in ageing is cellular senescence, a stress response of damaged or aged cells characterized by permanent cell cycle arrest.^313^ Like senescence, autophagy is a pathway that is activated upon cellular stress, and both pathways appear connected. For instance, oncogenic HRAS triggered senescence in fibroblasts that coincided with increased autophagy.^314^ Similarly, overexpression of p21 or ULK3 triggered both senescence and autophagy in fibroblasts.^314^, ^315^ Further, the inhibition of autophagy suppressed irradiation‐induced senescence, collectively suggesting that autophagy positive regulates senescence.^316^ On the contrary, knockdown of ATG5 or ATG7 in human fibroblast cells or ATG7 in murine muscle stem cells also induced senescence.^317^, ^318^ Further, GATA4, a senescence regulator, was found to be selectively broken down via p62‐mediated autophagy, which was repressed when cells were undergoing senescence, suggesting that autophagy and senescence repress each other’s activity.^319^ However, future experiments will have to elucidate whether autophagy can directly influence senescence or whether it is a co‐occurring pathway and whether autophagy modulation may be used to regulate senescence.^320^

Based on the evidence reviewed here, the inhibition of autophagy in not only cancer cells but also in cancer‐supporting stromal cells may help increase cancer cell death, especially in combination with other therapeutic approaches. Indeed, several phase I/II clinical trials are currently being conducted in various cancers with established autophagy inhibitors, such as 3‐MA, CQ, or HCQ (Table 1). On the contrary, an effective anticancer immune response appears to require autophagy at multiple levels. For instance, autophagy promotes the generation of antigenic peptides to be presented in MHC‐I and MHC‐II molecules on APCs. Further, T‐cell rely on an active autophagy pathway for their proliferation and survival. Hence, systemic application of autophagy inhibitors would likely inhibit anticancer immune responses. Indeed, the in vitro treatment of T‐cell with CQ reduced T‐cell–dependent cell lysis, inhibited T‐cell proliferation and reduced cytokine secretion.^321^ Further, accumulation of autophagic vacuoles was observed in PBMCs of patients treated with HCQ, suggesting that inhibition of autophagy occurred in these immune cells.^21^, ^22^, ^23^, ^322^ In line with this, the inhibition of autophagy was associated with lymphopenia in 31% of the patients in the temsirolimus study, whereas autophagy activation using mTOR inhibitor everolimus negatively affected multiple immune cell subsets in renal carcinoma patients.^21^, ^22^ Thus, autophagy inhibition likely occurs with current therapeutics, but should be avoided in cancer‐associated immune cells.

**Table 1:** Overview of phase I/II clinical trials in various cancers with established autophagy inhibitors. The table indicates the number of patients, treatment approach, patient outcome and the impact on autophagy activity

Malignancies	Patient numbers	Treatment	Achievement/outcome	Autophagic response	References
• Non–small‐cell lung cancer	8	HCQ	Dose‐limiting toxicity: not determined Maximum dose tolerance: not determined Outcome: progressive disease median PFS 1.8 mo, median OS 9 mo	Autophagic changes: not determined	Goldberg et al, A phase I study of erlotinib and HCQ in advanced non‐small cell lung cancer. *J Thorac Oncol*. 2012
	19	HCQ + erlotinib	Dose‐limiting toxicity: none Maximum dose tolerance: 1000 mg/d Recommended phase II dose: 1000 mg/d Outcome: PR, SD, median PFS 2 mo, median OS 10.6 mo	Autophagic changes: not determined	
• Colon rectal • Non–small‐lung cancer • Ovarian • Soft tissue Sarcoma • Renal • Breast • Melanoma • Carcinoid • Bladder • Prostate	27	Vorinostat (HDACi) + HCQ	Dose‐liming toxicity: 800 mg/d HCQ Maximum dose tolerance: 600 mg/d HCQ Outcome prolonged SD, PR	Autophagic changes: no significant changes in AV accumulation at Day 49 IHC revealed increased MAP1LC3B at Day 49	Mahalingam et al, Combined autophagy and HDAC inhibition/*Autophagy*. 2014
• Melanoma • Colorectal • Head and neck • Breast • Gastric/esophageal • Prostate • Pancreas • Non–small‐cell lung • Pheo/adrenocortical	27	Temsirolimus (mTOri) + HCQ	Dose‐limiting toxicity: 1200 mg/d HCQ Maximum dose tolerance: 1200 mg/d HCQ for 3 mo, subsequently lowered to 1000 mg/d HCQ Outcome: PFS of melanoma patients 3.5 mo, SD, no response	Autophagic changes: with 1200 mg/d HCQ significant AV accumulation at 6 wk, increased AVs with temsirolimus + HCQ compared to single HCQ treatment	Rangwala et al, Combined MTOR and autophagy inhibition. *Autophagy*. 2014
• Non–small‐cell lung cancer • Head and neck • Melanoma • Colon • Breast • Liposarcoma • Esophageal (SCC) • Brain metastasis	37	Temozolomide + HCQ	Dose‐limiting toxicity: none Maximum dose tolerance: not reached Recommended Phase II dose: 1200 mg/d Outcome: melanoma and breast PFS > 4 mo, PR, SD	Autophagic changes: accumulation of AVs at 4 wk	Rangwala et al, Phase I trial of HCQ with dose‐intense temozolomide in patients with advanced solid tumors and melanoma. *Autophagy*. 2014
• Glioblastoma multiforme	16	Radiation therapy + temozolomide + HCQ	Dose‐limiting toxicity: 800 mg/d HCQ Maximum dose tolerance: 600 mg/d HCQ	Autophagic changes: not determined	Rosenfeld et al, A phase I/II trial of HCQ in conjunction with radiation therapy and concurrent and adjuvant temozolomide in patients with newly diagnosed glioblastomas multiforme. *Autophagy*. 2014
	76	Radiation therapy + temozolomide + HCQ	Outcome: median OS 15.6 mo	Autophagic changes: increased AVs/cell and LC3‐II/I at 3 wk	
• Relapsed/refractory myeloma	25	Bortezomib + HCQ	Dose‐limiting toxicity: none Maximum dose tolerance: 1200 mg/d HCQ Outcome: VGPR, MR, SD, and immediate progression	Autophagic changes: increased AV and LC3‐II/I conversion (2 and 3 wk after treatment, respectively)	Vogl et al, Combined autophagy and proteasome inhibition. *Autophagy*. 2014
• Metastatic pancreatic adenocarcinoma	20	HCQ	Dose‐limiting toxicity: not determined Maximum dose tolerance: not determined Outcome: Median PFS 45.6 d, overall survival 69 d	Autophagy changes: inconsistent autophagy inhibition	Wolpin et al, Phase II and PD study of autophagy inhibition using HCQ in patients with metastatic pancreatic adenocarcinoma. *The Oncologist*. 2014
• Pancreatic adenoma	35	HCQ + gemcitabine + surgery	Dose‐limiting toxicity: none Maximum dose tolerance: 1200 mg/d HCQ Outcome: median DFS 12.0 mo	Autophagic changes: end of treatment 65% of patients showed increased LC3‐II staining, which correlated with improved DSF and OS	Boone et al, Safety and biologic response of preoperative autopagy inhibition in combination with Gemcitabine in patients with pancreatic adenocarcinoma. *Ann Surg Oncol*. 2015
Sarcoma	Closed early	HCQ + sirolimus	Dose‐limiting toxicity: not determined Maximum dose tolerance: not determined Outcome: PR, SD, progressive disease, no reduction of tumor volume	Autophagic changes: not determined	Chi et al, Double autophagy modulators reduce 2‐deoxyglucose uptake in sarcoma patients. *Oncotarget*. 2015
• Advanced metastatic BRAF mutant melanoma	11	Debrafenib (BRAFi) + trametinib (MEKi) + HCQ	Dose‐limiting toxicity: not determined Maximum dose tolerance: not determined Outcome: low rate retinal toxicity	Autophagic changes: not determined	Nti et al, Frequent subclinical macular changes in combined BRAF/MEK inhibition with high‐dose HCQ as treatment of advanced metastatic BRAF mutant melanoma. *Retina*. 2017
Relapsed/refractory multiple myeloma	6	Rapamycin + cyclophosphamide + dexamethasone	Dose‐limiting toxicity: none Maximum dose tolerance: not determined Outcome: MR, SD	Autophagic changes: not determined	Scott et al, Double autophagy stimulation using chemotherapy and mTOR inhibition combined with HCQ for autophagy modulation in patients with relapsed or refractory MM. *Haematologica*. 2017
		HCQ + cyclophosphamide + dexamethasone	Dose‐limiting toxicity: 1200 mg/d HCQ Maximum dose tolerance: not determined Outcome: PR, SD	Autophagic changes: not determined	
	18	Rapamycin + HCQ + cyclophosphamide + dexamethasone	Dose‐limiting toxicity: 1200 mg/d HCQ Maximum dose tolerance: 800 mg/d HCQ Outcome: VGPR, PR, MR, SD, immediate progression, median PFS 8.6 mo, median OS 11.3 mo	Autophagic changes: increased AV counts in 600‐1200 mg/d HCQ	

Abbreviations: 3‐MA, 3‐Methyladenine; CQ, chloroquine; HCQ, hydroxychloroquine; AV, autophagic vacuoles; DFS, disease‐free survival; IHC, immunohistochemistry; MR, minor response; OS, overall survival; PFS, progression‐free survival; PR, partial response; SD, stable disease; VGPR, very good partial response.

Of note, in a phase II study in glioblastoma patients, 600 mg/d HCQ was found to be the maximum tolerated dose, a dose at which autophagy inhibition was not consistently achieved in the tumor.^323^ Doses used in other early clinical cancer studies range from 800 to 1200 mg/d (Table 1). Thus, the therapeutic window for the clinical use of CQ seems to be quite small. Indeed, as also recently reported by us, there is only a small therapeutic window of autophagy inhibition with HCQ between CD34^+^ AML cells and healthy normal bone marrow‐derived CD34^+^ cells (Figure 3D).^108^ Moreover, current autophagy inhibitors such as CQ have a poor biodistribution profile, with levels of CQ in the blood being much higher than in the tumor.^324^

Taken together, it is clear that development of therapeutic strategies that inhibit autophagy more selectively in cancer cells appears warranted. Hereto, the development of novel autophagy inhibitors that have an increased activity profile in vivo, with limited cytotoxicity may help improve the therapeutic window for autophagy inhibition. For instance Lys05, a bisaminoquinoline and synthetic derivative of CQ was 10‐fold more potent than HCQ through better accumulation and deacidification of lysosomes and was effective in inhibiting autophagy in xenograft studies.^325^ Another inhibitor of interest is ARN5187, which blocks the final step of autophagolysosome maturation, with superior cytotoxic activity over HCQ in various cancer cell lines.^326^ Several other approaches to inhibit autophagy that target upstream regulatory events are currently under investigation in leukemia and solid cancer treatment. These include the PI3K inhibitor buparlisib, which selectively targets VPS34.^327^ First promising results in phase I single‐agent studies were observed in solid cancers as well as advanced leukemias, although toxicity was detected.^328^, ^329^ Other upstream targets being investigated include ULK.^330^, ^331^, ^332^ One of the more potent inhibitors of ULK1, SBI‐0206965, had prominent anticancer activity in human non–small‐cell lung cancer and adenocarcinoma.^332^, ^333^ All of these new autophagy‐targeting drugs are in early stages, with future clinical trials being awaited to gain insight into toxic effects and potential anticancer efficacy.

Further, increasing the tumor selectivity of autophagy drugs can be pursued, eg by use of liposomal formulations. Such formulations have been widely used to enhance drug retention and alter biodistribution by passive or active targeting. In this respect, encapsulation of chemotherapeutic drugs such as doxorubicin in liposomes has yielded more effective formulations with less side effects.^334^ Such formulations can be further optimized by using antibody‐conjugated and tumor‐targeted liposomes.^335^ Of note, CQ liposomes have already been generated in the context of CQ as a malaria drug and were also suitable to simultaneously deliver CQ and a tumoricidal drug.^336^, ^337^, ^338^, ^339^, ^340^, ^341^ The codelivery of CQ and doxorubicin improved the anticancer activity compared with liposomal doxorubicin.^341^ Alternatively, drugs such as CQ may be directly targeted to cancer cells using antibody‐drug conjugates.^342^

Future studies will have to determine whether selective targeting of cancer cells can prevent the adverse effect of inhibition of anticancer immunity. Concerning in this respect is the reported upregulation of PD‐L1 upon inhibition of autophagy, which results in dampening of immune responses. If further proven, combined treatment with autophagy and PD‐1/PD‐L1 checkpoint inhibitors may prove a straight‐forward approach to circumvent this issue. Nevertheless, inhibition of autophagy also alters the composition of peptides presented in MHC and thus may still impact on cancer immunity. Studies that elucidate this potential effect of autophagy inhibition in more detail are urgently needed to steer design of future clinical studies.

As it is evident that autophagy can steer the pool of antigenic peptides that are presented in MHC‐I and MHC‐II on the cell surface, attempts have been made to exploit autophagy for the purpose of therapeutic vaccination. For instance, fusion of NY‐ESO‐1, a cancer‐testis antigen frequently overexpressed in melanoma, to LC3 resulted in autophagosome targeting and augmented NY‐ESO‐1‐specific antimelanoma helper T‐cell responses.^343^ An additional approach to exploit autophagy for induction of immunity is through autophagosome‐based vaccination. Here, autophagosomes are isolated from cancer cells treated with a proteasome inhibitor. Such autophagosomes contain tumor‐associated antigens and on the surface express CLEC9A ligands that facilitate endocytosis by APCs.^344^ DCs pulsed with such autophagosomes were more efficient in inducing OVA‐specific T‐cell responses compared with soluble protein.^345^, ^346^ This autophagosome vaccination approach reduced B16F10 melanoma cell growth, eliminated 3LL Lewis lung tumors, and protected mice from a rechallenge with sarcoma.^346^, ^347^ Of note, tumor cells may also release autophagosomes themselves and modulation of autophagy could, therefore, trigger the release of autophagosomes from tumor cells and positively impact on anticancer T‐cell immune responses.^280^, ^348^

Of note, an important issue that needs to be addressed for any new type of therapy to enter clinical practice is the identification of appropriate patient stratification criteria. In this respect, current clinical trials do not have inclusion/exclusion criteria that take autophagic activity in the tumor into account. Further, appropriate testing of the efficacy of autophagy inhibition in cancer cells of patients will have to be developed, with current trials mainly monitoring autophagy in peripheral blood mononuclear cells (PBMCs) as a surrogate marker of response or in tumor biopsies. However, the level of autophagy in PBMCs does not seem to correlate with autophagy inhibition in the tumor microenvironment.^23^ Therefore, positron emission tomography/computed tomography and magnetic resonance imaging probes for ATG activity are currently being developed. Thus, research in the upcoming years should focus on not only identifying optimal inhibitors of autophagy in patients, but also on the identification of appropriate patient selection criteria and monitoring tools to position autophagy targeting for clinical use.

In conclusion, a host of evidence has emerged on the importance of autophagy in cancer cells and its validity as a target for this disease. However, the various cell types in the tumor microenvironment differ in their reliance on autophagy, making it hard to predict the exact outcome of autophagy inhibition in cancer. Further detailed investigations into the specific impact in this complex milieu are needed to steer rational design of therapeutic targeting of autophagy in specific cancer subtypes and combination strategies.
